# The Fe-MAN Challenge: Ferrates–Microkinetic Assessment of Numerical Quantum Chemistry

**DOI:** 10.1021/acs.jpca.4c01361

**Published:** 2024-06-04

**Authors:** Rene Rahrt, Björn Hein-Janke, Kosala N. Amarasinghe, Muhammad Shafique, Milica Feldt, Luxuan Guo, Jeremy N. Harvey, Robert Pollice, Konrad Koszinowski, Ricardo A. Mata

**Affiliations:** † Institut für Organische und Biomolekulare Chemie, Universität Göttingen, Tammannstr. 2, Göttingen 37077, Germany; ‡ Institut für Physikalische Chemie, Universität Göttingen, Tammannstr. 6, Göttingen 37077, Germany; § Leibniz Institute for Catalysis (LIKAT), Albert-Einstein-Str. 29A, Rostock 18059, Germany; ∥ Department of Chemistry, KU Leuven, Celestijnenlaan 200F, Leuven B-3001, Belgium; ⊥ Stratingh Institute for Chemistry, University of Groningen, Nijenborgh 4, Groningen 9747 AG, The Netherlands

## Abstract

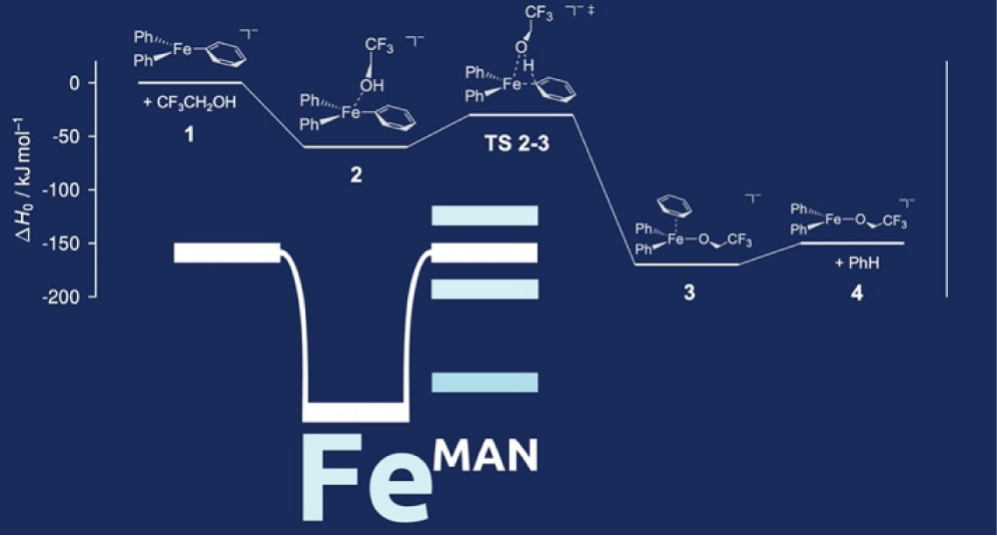

Organometallic species, such as organoferrate ions, are prototypical nucleophiles prone to reacting with a wide range of electrophiles, including proton donors. In solution, the operation of dynamic equilibria and the simultaneous presence of several organometallic species severely complicate the analysis of these fundamentally important reactions. This can be overcome by gas-phase experiments on mass-selected ions, which allow for the determination of the microscopic reactivity of the target species. In this contribution, we focus on the reactivity of a series of trisarylferrate complexes toward 2,2,2-trifluoroethanol and 2,2-difluoroethanol. By means of mass-spectrometric measurements, we determined the experimental bimolecular rate constants *k*_exp_ of the gas-phase protolysis reactions of the trisarylferrate anions FePh_3_^–^ and FeMes_3_^–^ with the aforementioned acids. Based on these experiments, we carried out a dual blind challenge, inviting theoretical groups to submit their best predictions for the activation barriers and/or theoretical rate constants *k*_theo_. This provides a unique opportunity to evaluate different computational protocols under minimal bias and sets the stage for further benchmarking of quantum chemical methods and data-driven approaches in the future.

## 1 Introduction

The Fe-MAN (Ferrates–Microkinetic Assessment of Numerical quantum chemistry) challenge is a dual blind challenge aimed at the critical assessment of theoretical predictions in gas-phase kinetics. In a dual blind-challenge, theory predictions are submitted for a set of unknown observables. The experimental group carrying out the measurements is also unaware of the aforementioned predictions. In the present case, theoreticians were given the challenge of submitting reaction barriers and/or kinetic rate constants for selected reactions. With the increasing accessibility of computational methods and the plethora of approaches available, benchmarking has become a common practice. However, this is usually carried out only by individual groups and not in a concerted fashion across the community. Yet, a few exceptions exist.^1−5^ The choice of focusing on kinetics is not a casual one but a conscious decision to diversify the types of observables to be benchmarked against. Benchmarking in the gas phase has been previously centered on evaluating noncovalent interactions, as these are easier to address by experiments.^6^ In a joint effort between experiment and theory, the Fe-MAN challenge was set up and organized, following the example of previous challenges.^7−9^ Importantly, to the best of our knowledge, this is the first of its kind looking into kinetics.

The benefits of such community efforts are manifold. Foremost, one has a unique opportunity to evaluate computational procedures in an unbiased way. The Pauling point is an expression that has come to be less used nowadays but is used to highlight how theoreticians often only go so far with their computations to the point where agreement with experimental values/observations could be found. In the words of Per Lowdin, “even a fairly simple theory could sometimes give excellent agreement with experimental experience, but this agreement may disappear whenever one tries to improve the theory. The point of excellent agreement was coined the Pauling point”.^10^ In other words, it is the point where one reaches the right result, even if not all of the physics of the problem are included. This, of course, is possible only with prior knowledge of the target value. A blind challenge removes this possibility altogether. Depending on the choice of system and quantity under study, error compensation is still possible but can no longer be engineered.

The other benefits come after the challenge itself. The experimental data acquired can be reused over the years to further test and guide the development of quantum chemical approaches, even if the blind factor is removed. Furthermore, this opens the door for some systematic investigations of the computational protocols. As one will observe, there is some heterogeneity in the submitted theoretical works. Each group picked their favorite approach(es) to the problem, and there is no extensive evaluation of the protocols (basis set size, optimization method, conformational sampling approach, ...).^11^ The limited time for this challenge gives little leeway for these assessments. In this publication, we try to consider, as much as possible, some individual factors, but this is limited by the data offered by each participating group. Despite this, the work sets the stage for follow-up investigations on how calculations can converge toward experiments.

In this contribution, we set out the challenge for research groups to predict barriers or rate constants of protonation reactions of arylferrate anions with alcohols in the gas phase. Protonation is one of the prototypical reaction modes of organometallics. Furthermore, this also corresponds to an example of a reaction between a nucleophile and an electrophile. As protonation is the simplest electrophile conceivable, protonation reactions lend themselves particularly well as models to study the influence of electronic and steric effects on the reactivity of organometallics. Nonetheless, the analysis of such reactions in solution is notoriously difficult due to the operation of complex dynamic equilibria and the simultaneous presence of different organometallic species, which can be expected to differ in their individual reactivity. To solve this problem and determine the microscopic reactivity of a series of well-defined arylferrate complexes, we probed their gas-phase reactions in a quadrupole-ion trap (QIT).^12^ This instrument permits the selection of ions of a given mass-to-charge (*m*/*z*) ratio and thereby excludes any interference by dynamic equilibria. Some of us (RR and KK) have recently demonstrated the success of this approach for the gas-phase protonation of organozincate anions.^13,14^ The measured bimolecular rate constants could be reproduced by theoretical calculations within a factor of 8 and thus served as a valuable benchmark for the latter. In contrast to zinc, the iron present in the complexes probed here features an open shell of d electrons and therefore poses a significantly greater challenge to theoretical calculations. With all of the inherent difficulties in experimentally assessing the kinetic rate constants, there is also a lack of reference data upon which modelers can train/develop their approaches. This is particularly severe for data-driven methods.

**Scheme 1:**
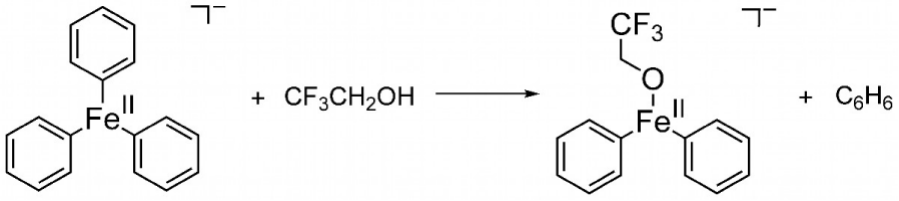
Training Reaction used in the Challenge, Featuring FePh_3_^–^ as the Reactant Ion and R^F3^OH as the Reacting Alcohol

From the contributions of four different groups, we have 7 sets of theoretical estimates for the 5 test reactions (see Table 1 and Scheme S1). A training reaction (Scheme 1) was also provided for the participants before the challenge. The corresponding rate constant was made known, together with data from analogous experiments on trisarylzincates.^13^ This allowed each participating group to test their approaches on related systems beforehand.

**Table 1:** Investigated Protolysis Reactions of the Trisarylferrate Anions with the Alcohols ROH^a^^,^^c^

reaction^a^	reactant ion^b^^,^^c^	alcohol^c^	product ion^b^^,^^c^
0	FePh_3_^–^	R^F3^OH	FePh_2_(R^F3^O)^−^
1	FePh_2_(OR^F3^)^−^	R^F3^OH	FePh(R^F3^O)_2_^–^
2	FePh_3_^–^	R^F2^OH	FePh_2_(R^F2^O)^−^
3	FePh_2_(OR^F2^)^−^	R^F2^OH	FePh(R^F2^O)_2_^–^
4	FeMes_3_^–^	R^F3^OH	FeMes_2_(R^F3^O)^−^
5	FeMes_2_(OR^F3^)^−^	R^F3^OH	FeMes(R^F3^O)_2_^–^

a Reaction 0 served as training data, reactions 1–5 as the test reactions.

b Mes = mesityl.

c R^F3^OH = CF_3_CH_2_OH, R^F2^OH = CF_2_HCH_2_OH.

After the test reactions had been announced, the research groups were given four and a half months to submit their predictions. None of the groups were provided with experimental data on the test systems, besides the expectation that all complexes were in a high-spin state. The assumption was based on experimental evidence of similar systems from condensed-phase studies.^15,16^ Following the deadline, the submissions were reviewed, and where necessary, further data was requested. The individual submitted theoretical datasets are explained later in the text (Table 2). Following the publication of the experimental rate constants, none of the groups were allowed to change their submissions. The procedure is similar to previous blind challenges referencing experimental data.^7,8^

**Table 2:** Summary of Computational Entries, which are Critically Compared in this Work^a^

entry	structure optimization	electronic single-point energies	kinetic model
**A**	ωB97X-D3/def2-TZVP	LUCCSD(T)/def2-TZVP	microcanonical; ME
**B**	ωB97X-D3/def2-TZVP	DLPNO-CCSD(T)/def2-TZVP	microcanonical; ME
**C**	ωB97X-D3/def2-TZVP	PNO-LCCSD(T)-F12/def2-TZVP	microcanonical; ME
**D**	BP86-D3(BJ)/def2-SVP	[B3LYP-D3(BJ) + PBE0-D3(BJ)]/def2-TZVP	microcanonical; ME
**E**	B3LYP-D3(BJ)/def2-SVP	B3LYP-D3(BJ)/def2-TZVPD	microcanonical; ME
**F**			canonical; TST
**G**	data-driven model		

a ME stands for the Master equation calculations. The E and F entries are based on the same set of calculations but differ in the way the theoretical rate constants were derived.

We start by reviewing the experimental investigations that supported the challenge and discussing the derived kinetic data. Subsequently, we critically analyze both the experimental methodology and the theoretical submissions and, on this basis, identify future challenges.

## 2 Methods

### 2.1 Experimental Methods

We determined the experimental bimolecular rate constants *k*_exp_ of the gas-phase protolysis reactions of the trisarylferrate anions FePh_3_^–^ and FeMes_3_^–^ (Mes = mesityl) by the proton donors 2,2,2-trifluoroethanol (R^F3^OH) and 2,2-difluoroethanol (R^F2^OH) at *T* = (310  ± 20) K^17,18^ following the previously described methodology.^13,14^

#### 2.1.1 Preparation of Sample Solutions

Sample solutions were prepared under standard inert-gas conditions. THF was dried over sodium benzophenone and freshly distilled. All other chemicals were purchased from Sigma-Aldrich and used without further purification. A solution of Fe(acac)_3_ (0.035 g; acac = acetylacetonate) in tetrahydrofuran (THF, 10 mL; 10 mm) was cooled to 195 K and treated with solutions of PhMgCl (4 equiv) or MesMgBr (4 equiv).

#### 2.1.2 Mass-Spectrometric Measurements

The sample solutions were injected into the electrospray-ionization (ESI) source of a QIT-mass spectrometer (HCT, Bruker Daltonik, Bremen, Germany) by pressurized sample infusion^19^ at 195 K. The ESI capillary voltage was set to 3000 V. Nitrogen was used as a nebulizer (0.7 bar) and dry gas (333 K, 5.0 L min^–1^). The trap drive of the QIT was set to 33.5.

To conduct kinetic measurements of the gas-phase reactions, the ions of interest were mass-selected and stored in a QIT (MS*^n^*). Therein, they reacted with the substrate gases R^F3^OH (25, 50, or 100 μL) or R^F2^OH (100 μL), which were introduced *via* a home-built gas-mixing and inlet apparatus.^20^ To monitor the time dependence of the investigated protolysis reactions, the storage time *t* was varied (0 to 5000 ms). MS*^n^* mass spectra were recorded for at least 1 min for each time interval and averaged with DataAnalysis by Bruker Daltonik.

#### 2.1.3 Determination of Experimental Rate Constants

The signal intensities of the reactant and product ions were extracted and normalized (product ions with signal intensities below 5% were neglected). For each time step, the normalized signal intensities were plotted against the reaction time *t*. The kinetic profiles were then fitted according to the given reaction networks (Schemes S2–S4) with the program GEPASI 3.30^21−23^ to afford the effective rate constants *k*_eff_. These pseudo-first order rate constants were converted into the bimolecular rate constants *k*_exp_ by dividing them by the known substrate concentration *N*_substrate_/*V* in the QIT (R^F3^OH: 4.3×10^10^–1.7×10^11^ cm^–3^; R^F2^OH: 1.8×10^11^ cm^–3^).

Each kinetic measurement was conducted in at least two independent experiments. The given experimental uncertainty corresponds to the relative error of the statistical deviation (95% confidence interval). The absolute error is estimated to be ±30% due to the uncertainty of the partial pressure of the substrate.^20^ Reaction efficiencies *φ* were determined by dividing the experimental rate constant *k*_exp_ by the collision rate *k*_coll_, which was estimated according to the capture theory of Su and Chesnavich (Table S2).^24−26^

### 2.2 Computational Methods

#### 2.2.1 Quantum-Chemical Calculations

The participants of the Fe-MAN challenge employed different quantum-chemical methods for the conformational search and optimization of stationary points, including reactants, intermediates, transition states, and products. Electronic single-point energy calculations were used to refine the energetics values.

In the following section, we give a short overview of the different entries. Further computational details can be found in the Supporting Information for each submission. Entries **A**–**C** have been submitted by the same group. They are based on structures optimized at the ωB97X-D3/def2-TZVP^27−29^ level of theory. Subsequently, three sets were generated, differing only in the single-point energy refinement used. Entry **A** makes use of PAO-based local unrestricted coupled cluster singles and doubles with perturbative triple excitations (LUCCSD(T)).^30^ Entry **B** makes use of domain-based local pair natural orbital coupled cluster (DLPNO-CCSD(T)),^31−34^ while Entry **C** resorts to pair natural orbital coupled cluster with explicit correlation (PNO-LCCSD(T)-F12).^35−40^ All of the aforementioned single-point calculations made use of the def2-TZVP basis set. The three entries allow for a critical evaluation of the performance of the different coupled cluster variants. For entries **A**–**C**, no values are provided for reaction 4 due to difficulties in obtaining the respective structures.

In the case of entry **D**, the structures were obtained at the BP86-D3(BJ)/def2-SVP level of theory.^41−44^ The training system was then used for a reparameterization of both B3LYP-D3(BJ)^45−47^ and PBE0-D3(BJ)/def2-TZVP.^48,49^ The amount of exact exchange was varied until the estimated barrier was consistent with the experimental data. By doing so, both functionals provide the same value of 30 kJ mol^–1^ for the barrier in reaction 0. Using these reparametrized functionals, single-point calculations were carried out for the test systems. The average of the two DFT results was used as a prediction, and the deviation between the two was used to estimate the error. The deviation signals a failure in the parametrization, which is the reason why it was taken as an error bar.

Submissions **E** and **F** stem from the same group. The stationary points are obtained at the same level of theory, B3LYP-D3(BJ)/def2-TZVPD//B3LYP-D3BJ/def2-SVP. However, in this case, the proponents did not only compute the energetics but also predicted rate constants based on a canonical model (entry **F**), taking into account multiple conformers of the transition state, and also accounting for the degeneracy of reaction paths through a “symmetry factor”.^50^ Upon discussions with the group, it was proposed to run Master-equation calculations based on their quantum chemical results for only the lowest-lying conformer of the TS to each reaction (entry **E**), so that a more direct comparison to the other datasets could be made.

It should be noted that for all entries **A**–**F** a lower level of theory was applied to find the transition state, with the final electronic energy being refined with a different (computationally more expensive) model chemistry. This was a pragmatic approach given the limited time provided for the challenge. None of the submissions included a reevaluation of the transition state position along the reaction coordinate (for example, with the use of the IRCMax procedure).^51^

In one case (submission **G**), rate constants were obtained from a data-driven model using linear free-energy relationships based on a very small set of experimental rate constants for the protolysis of comparable metal complexes (including other metals). For a summary of the entries, see Tables 2 and S1.

#### 2.2.2 Calculation of Theoretical Rate Constants

For the submissions **A**–**E,** the theoretical rate constants *k*_theo_ were obtained from Master-equation calculations as follows. As detailed above, submissions **F** and **G** provided rate constants *k*_theo_, which were determined following other methodologies (Tables 2, S1).

In order to determine theoretical rate constants by Master-equation calculations, the program MESMER by Glowacki and coworkers was used (settings: *ClassicalRotors*, *precision*: double-double, *grain size*: 20 cm^–1^, *simpleCalc*).^52^ The reaction pathways were simplified such that the reactants form the pre-reactive complex, which then directly reacts to the products via the transition structure. Consequently, stationary points between the TS and products were neglected in the kinetic modeling. This assumption is well justified, given that the proton transfer is the rate-determining step. The formation of the pre-reactive complex was computed with the implemented inverse Laplace transform (ILT) method based on the theoretical collision rate constants according to the capture theory by Su and Chesnavich (Table S2) for data sets **A**–**D**.^24−26^ For entry **E**, collision rates of 2.0 × 10^–9^ cm^3^ s^–1^ were assumed for all reactions to be consistent with the method followed in submission **F** (see Supplementary Information). The proton transfer step was modeled with RRKM theory. These calculations accounted for the reaction path degeneracy but did not consider multiple conformers of the transition state.

Using the computed rotational constants and vibrational frequencies of the reactants, pre-reactive complex and TS (for entry **E**, spurious imaginary frequencies or real frequencies smaller in magnitude than 50 cm^–1^ were replaced by real frequencies of 50 cm^–1^ to be consistent with submission **F**), and their respective enthalpies at 0 K, Δ*H*_0_, MESMER generated time-dependent species profiles for reaction conditions, which correspond to those in the experiment: *T* = 310 K, *p*_He_ = 0.6×10^–3^ mbar, *N*_substrate_/*V* = 4.3×10^10^ – 1.7×10^11^ cm^–3^ (R^F3^OH); 1.8×10^11^ cm^–3^ (R^F2^OH). The time-dependent species profiles were then fitted with GEPASI 3.30^21−23^ and pseudo-first-order rate constants were extracted and converted into the theoretical bimolecular rate constants *k*_theo_.^53^ It should be mentioned that in all entries, **A**–**F** symmetry has been factored in.

## 3 Results and Discussion

### 3.1 Experimental Results

**Figure 1:**
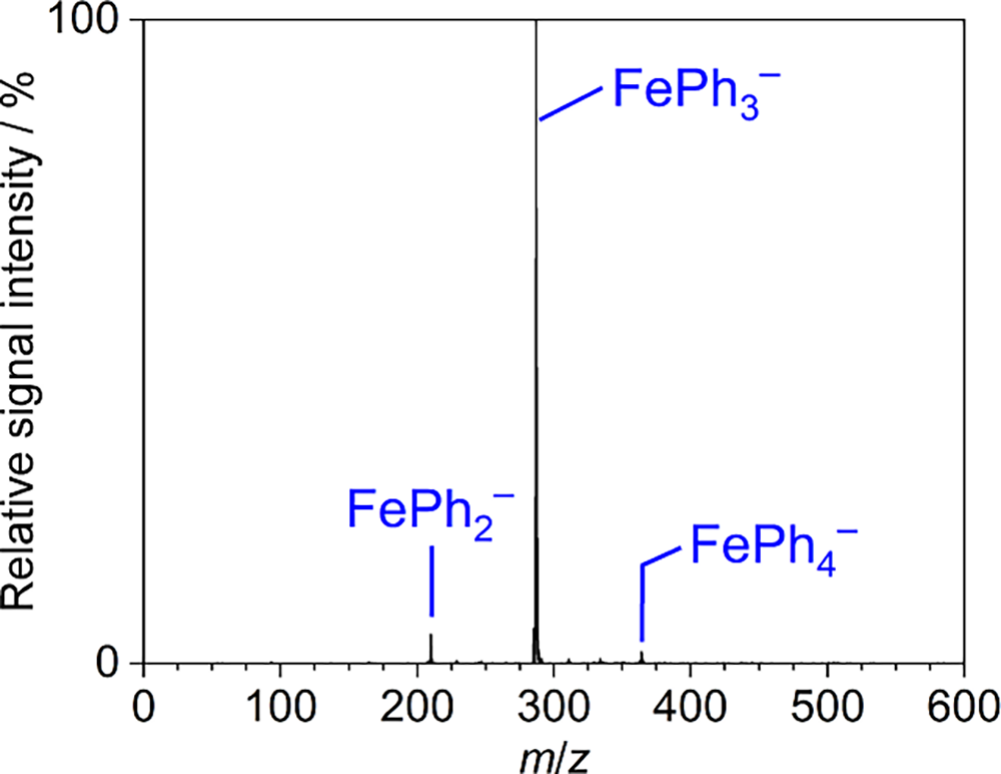
Negative-ion mode electrospray-ionization (ESI) mass spectrum (MS^1^) of the products formed from the reaction of Fe(acac)_3_ with PhMgCl (4 equiv) in THF at 195 K.

Gaseous trisphenylferrate, FePh_3_^–^, was readily prepared by electrospraying a solution of Fe(acac)_3_ and PhMgCl (4 equiv) held at 195 K. The negative-ion mode ESI-mass spectrum showed minor amounts of Fe(i)Ph_2_^–^ and Fe(iii)Ph_4_ aside from the dominant species Fe(ii)Ph_3_^–^ (Figure 1).

**Figure 2:**
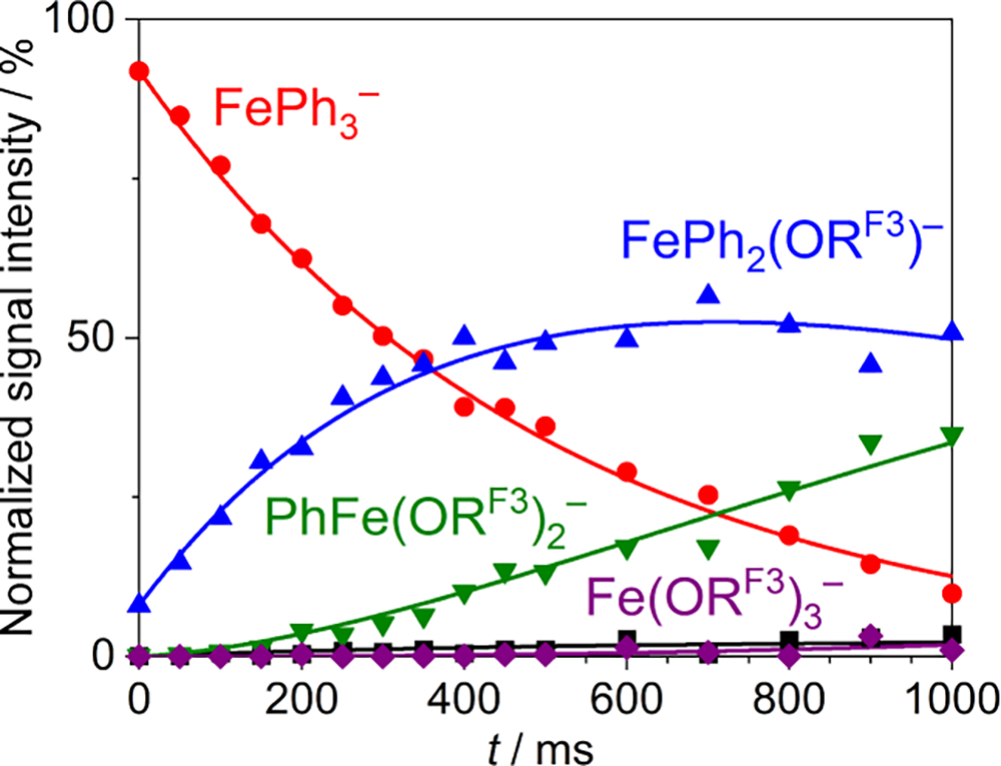
Time-dependent signal-intensity profiles (MS*^n^*) of mass-selected FePh_3_^–^ and the product ions resulting from its reaction with 2,2,2-trifluoroethanol (R^F3^OH). The fitting of the data gave pseudo-first-order rate constants, which were converted into the bimolecular rate constants *k*_exp_. The side reaction of FePh_3_^–^ with traces of formic acid (black squares) was accounted for in the kinetic modeling.

FePh_3_^–^ was mass-selected and subjected to a gas-phase ion-molecule reaction with R^F3^OH. Within a time of *t* = 1000 ms, the organoferrate was completely consumed in the protolysis reaction. First, trisphenylferrate FePh_3_^–^ and R^F3^OH reacted to give FePh_2_(OR^F3^)^−^ and benzene (reaction 0). Thereafter, the product of the first protonation, FePh_2_(OR^F3^)^−^, underwent another reaction with the proton donor R^F3^OH, which yielded FePh(OR^F3^)_2_^–^ and benzene (reaction 1). Moreover, the final protonation reaction in the protolysis sequence, in which FePh(OR^F3^)_2_^–^ was converted into Fe(OR^F3^)_3_^–^, and a side reaction of FePh_3_^–^ with traces of contaminant formic acid could be observed (Figure 2).

When analogous experiments were carried out at different partial pressures of R^F3^OH, the determined effective rate constants *k*_eff_ showed a linear correlation with the introduced amount of substrate *V*(R^F3^OH) (Figures S1 and S2). This behavior is in line with expectations and suggests that the experimental methodology indeed works as intended. The bimolecular rate constants determined for reactions 0 and 1 (Table 3) correspond to reaction efficiencies *φ* of 1.8% and 0.8%, respectively, thus indicating significant barriers for the protonation reactions.

**Table 3:**
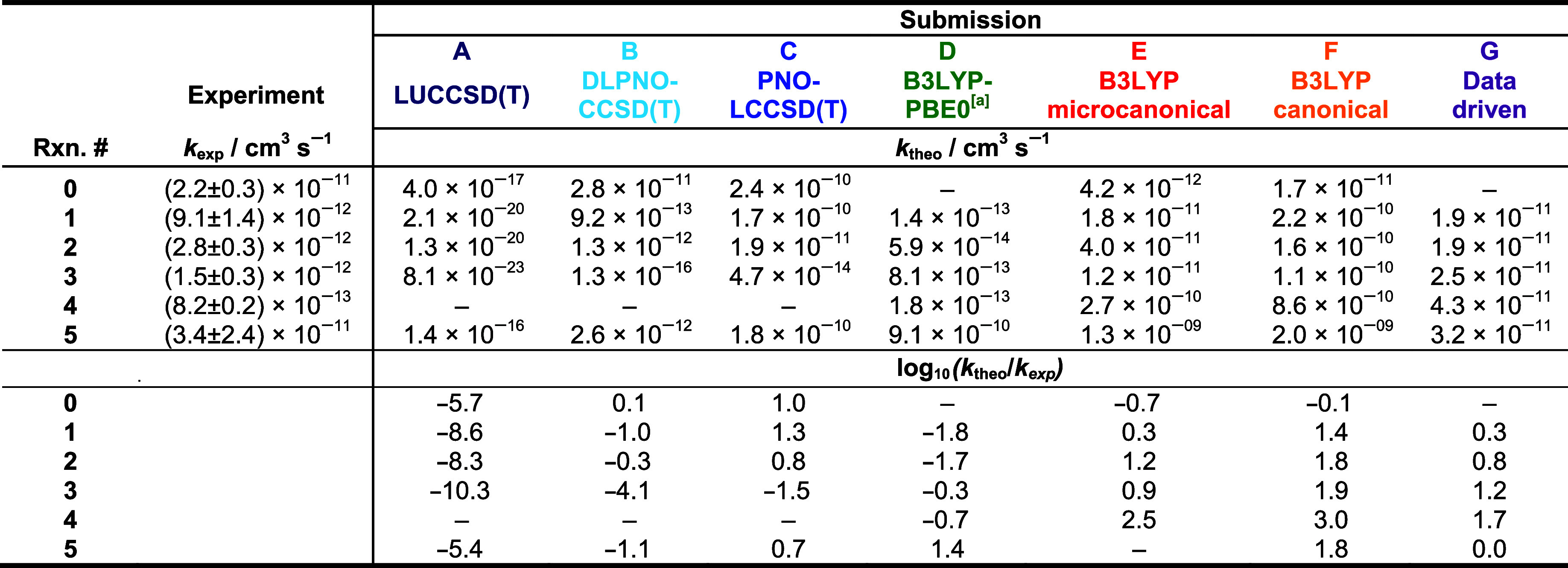
Compilation of the Experimental Rate Constants *k*_exp_, which were Determined from Gas-Phase Ion-Molecule Experiments, and the Theoretical Rate Constants *k*_theo_ as Obtained from the Challenge Participants or Determined from Master-Equation Calculations Based on the Participants’ Submitted Data^a^^,^^b^

a The given experimental uncertainty only reflects the relative errors (≤20%, 2σ). The absolute experimental uncertainty is larger (±30%). The ratio between theoretical and experimental rates is provided in log_10_ scale in the second half of the table.

b Minimum and maximum rate constants, *k*_min_ and *k*_max_, were determined from the uncertainties in the calculated energies of the prereactive complex and the TS. For the reactions #1 to #5 *k*_min_/cm^3^ s^–1^ = 2.5 × 10^–14^, 3.5 × 10^–14^, 2.5× 10^–13^, 3.4 × 10^–15^, 2.1 × 10^–10^ and *k*_max_/cm^3^ s^–1^ = 8.3 × 10^–13^, 1.0 × 10^–13^, 2.6 × 10^–12^, 9.9 × 10^–12^, 1.1 × 10^–9^, respectively.

Next, the tris(phenylferrate) FePh_3_^–^ reactions with the proton donor R^F2^OH were studied. Just as for R^F3^OH, three consecutive protonations affording Fe(OR^F2^)_3_^–^ as the final product ion were observed (Figure S3). However, the time required for the consumption of the reactant ions was longer than in the case of the protonation by R^F3^OH. Accordingly, the derived rate constants were lower, translating into reaction efficiencies of *φ* = 0.4% and 0.2% for reactions 2 and 3, respectively (Table 3). The kinetic analysis included side reactions, with traces of HCOOH and R^F3^OH remaining in the QIT.

The gaseous trismesitylferrate anion, FeMes_3_^–^, was prepared by electrospraying a solution of Fe(acac)_3_ and MesMgBr (4 equiv) at 195 K (Figure S4). When subjected to collisions with R^F3^OH, the mass-selected ion showed three consecutive protonation reactions. In marked contrast to the first two experiments, full conversion of the reactant ion was not achieved within a reaction time of *t* = 5000 ms. Even more interestingly, the product ion of the first protonation, i.e., FeMes_2_(OR^F3^)^−^ (reaction 4), was found in only small signal intensities. Apparently, the first protonation of FeMes_3_^–^ proceeded very slowly, but the second protonation (reaction 5) occurred so fast that FeMes_2_(OR^F3^)^−^ was consumed rapidly to give FeMes(OR^F3^)_2_^–^. Again, side reactions with traces of HCOOH took place and were taken into account in the kinetic analysis (Figure S5). The obtained rate constants correspond to reaction efficiencies of *φ* = 0.1% and 2.9% for reactions 4 and 5 (Table 3), respectively, and thus confirm that the second protonation proceeded much faster than the first one.

**Figure 3:**
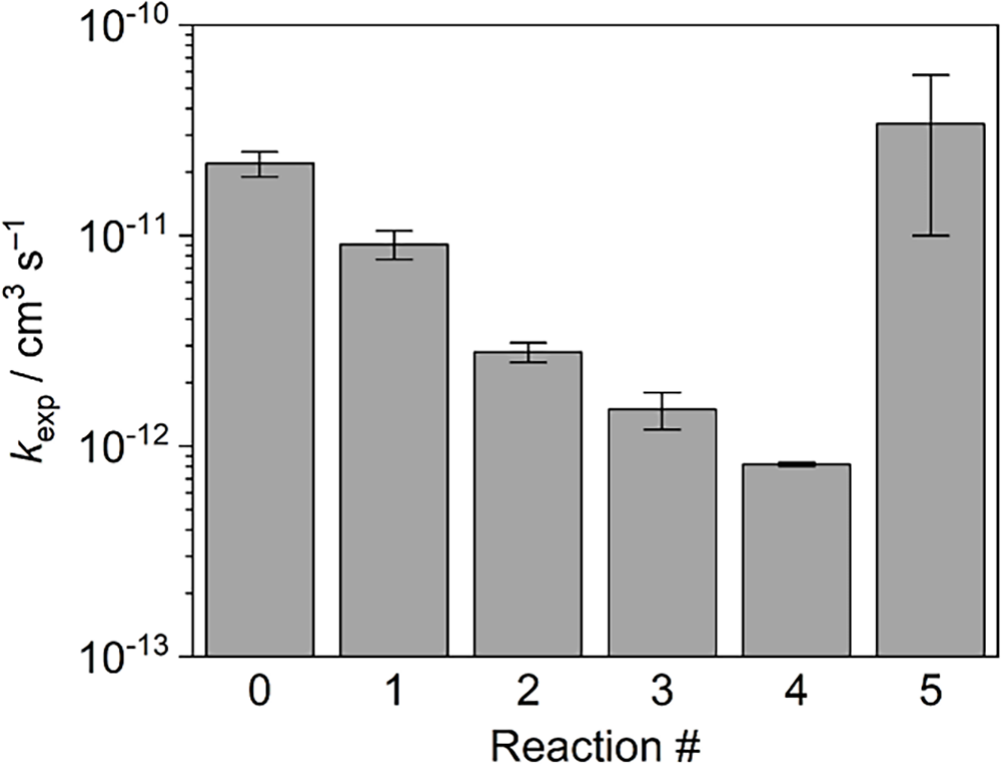
Experimental rate constants *k*_exp_ as obtained from the gas-phase ion-molecule reaction experiments for reactions 0–5. The error bars correspond to the statistical uncertainties (95% confidence interval).

The experimental rate constants *k*_exp_ for the reactions 0–5 increase in the order *k*(4) < *k*(3) < *k*(2) < *k*(1) < *k*(0) < *k*(5) (Figure 3). This ordering shows that the reactions with R^F3^OH as the proton donor (reactions 0, 1, 4, 5) are faster than those with R^F2^OH (reactions 2, 3). This trend is to be expected given that gaseous R^F3^OH is more acidic than R^F2^OH (Δ_acid_*G* = 1482 vs. 1503 kJ mol^–1^).^54^ Therefore, the reactions of the organoferrate anions with R^F3^OH are more exothermic, and thus, the protonation barrier will be reduced.

Furthermore, the second protonation reaction within a given protolysis sequence of FePh_3_^–^ is slower than the first one (more so than just expected from the decreased reaction-path degeneracy). Apparently, the first protonation deactivates the reactant ion for consecutive reactions. This deactivation presumably results from a decrease in the negative partial charges and basicities of the remaining phenyl moieties after the replacement of one phenyl by an alkoxy group. Being more electronegative than carbon, the oxygen atom of the alkoxy group abstracts more electron density from the iron center, which, in turn, donates less electron density to the carbon atoms of the phenyl moieties. In contrast, a similar trend does not hold for reactions 4 and 5. Here, the second protonation is much faster than the first one. The reason most probably lies in two opposing effects exerted by the mesityl moieties. First, the *ortho* substituents increase the size of the mesityl group and render the approach of the protonation donor more difficult. This effect predominates for FeMes_3_^–^, for which the presence of three mesityl groups effectively shields the reactive basic sites, thereby strongly diminishing the reactivity toward R^F3^OH (reaction 4). Second, the positive inductive effect of the methyl substituents increases the electron density and, thus, also the basicity of the mesityl groups. This electronic effect explains the reactivity enhancement observed for the second protonation step (reaction 5), when the replacement of the first mesityl group by an alkoxy ligand has opened efficient access to the reactive sites.

### 3.2 Theoretical Results

Figures S6–S11 depict the calculated rate constants, and Table 3 provides a comparison of the experimental rate constants *k*_exp_ to the theoretical rate constants *k*_theo_. It should be noted that the experimental uncertainties provided only reflect relative/statistical errors. The different sources of error in the experiment are discussed in further detail in the next section. Overall, most theoretical rate constants are within two orders of magnitude of the experimental value. This result might seem like a very modest degree of agreement, but it is generally observed that most computational protocols tend to lie in this accuracy range. This is particularly true when dealing with transition-metal complexes. In the case of trisarylzincates, it was possible to predict experimental rates within factors of 2–8 by making use of DFT-optimized structures, refining the energy with coupled cluster single-point calculations, and applying Master equation calculations.^13^ This observation is somewhat in line with entries **B** and **C,** although it can be argued that the atomic basis sets used were not sufficiently large. However, zinc is much easier to handle computationally than the iron metal center. The latter will exhibit some level of multireference character. This affects both DFT and wave function calculations. It should be noted that these are expected to be high-spin Fe^II^ species,^15,16^ and therefore not pathological multireference cases.

**Figure 4:**
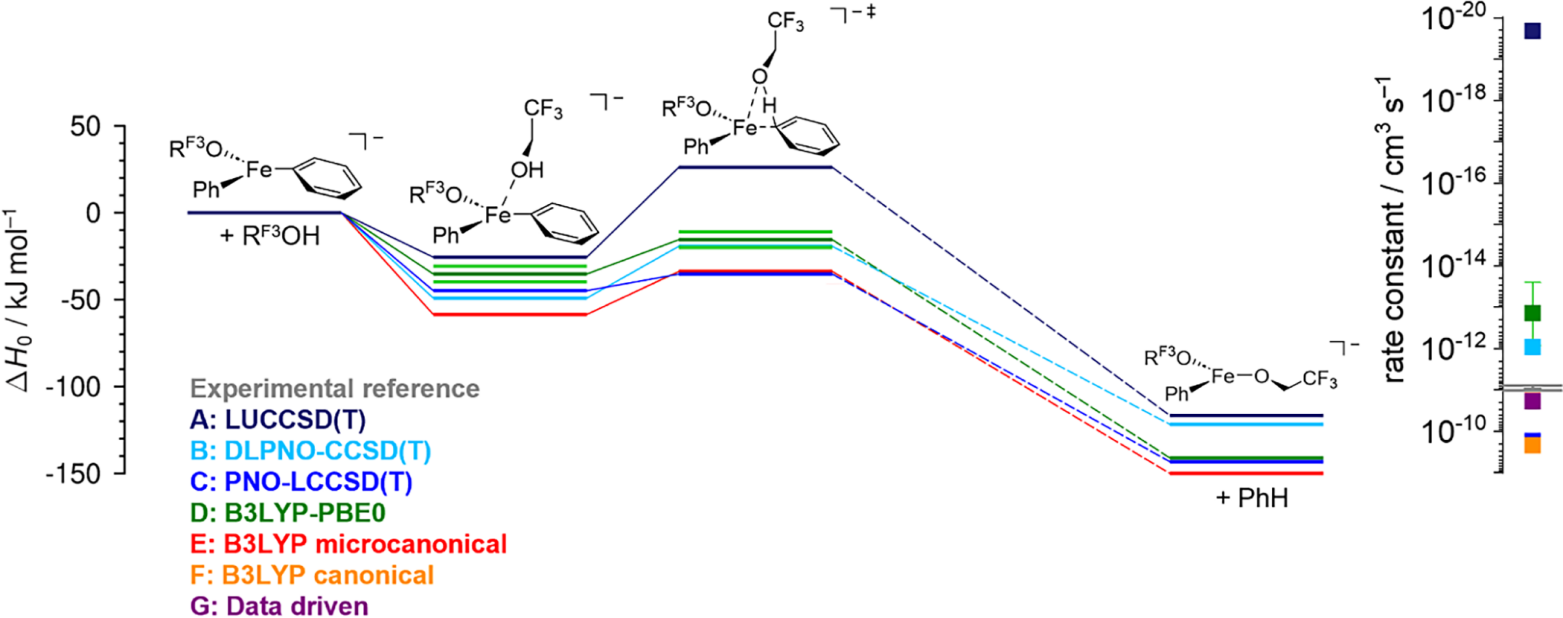
Energy profiles for reaction 1 of FePh_2_(R^F3^O)^−^ with 2,2,2-trifluoroethanol (R^F3^OH) as calculated by the denoted methods with the determined theoretical rate constants *k*_theo_ (colors) and the experimental rate constant *k*_exp_ as reference (gray).

Another way of comparing the different theoretical submissions is to plot the reaction energy profiles, such as in Figure 4. We consider as an example reaction 1. If the reactant molecules are taken as references in the energy scale, one can see that entry **A** is a clear outlier, crudely overestimating the barrier. Entries **B** and **D** (the latter given as a range) are in close agreement, while **C** and **E** predict a somewhat lower barrier. This is in itself useful information, but we are mostly concerned with the rates. Given the experimental conditions, we will focus on the values provided in the microcanonical regime. This will be the basis for evaluating the theoretical methods.

Regarding the rate constants, entry **A** shows the worst performance out of all theory sets. This is expected from observing Figure 4 and is likely linked to the domain error in the PAO-based LUCCSD(T) method. With small changes in the geometry, the orbital domains can vary, leading to discontinuities in the potential energy surface and potentially large errors in the relative energies.^55,56^ This error is greatly reduced when making use of pair natural orbitals (PNO)^57^ based approaches (entries **B** and **C**). Entries **B** and **C** are, in fact, among the top -performing submissions of this challenge, again showing the robustness of the CCSD(T) gold standard in quantum chemistry.

Entry **D** does not include values for reaction 0, given that the method was parameterized to exactly reproduce this reaction. Overall, this tends to underestimate the rate constants. Notably, DFT functionals generally tend to underestimate barriers (overestimate rates).^58^ Seeing the reverse trend might be an indication that the parametrization went too far and that the obtained functionals are not robust enough for this particular application. It is the only entry with an estimated error bar, with the largest error estimate in reaction 4. Again, this should be interpreted as the largest deviation between the two parameterized functionals. This is further discussed in the following section.

Comparing entries **E** and **F**, larger deviations are observed for **F**. In the latter case, the rate constants are overestimated (the reaction is predicted to be faster than what has been measured). Unfortunately, there are several differences between the two sets, to the point where it is hard to exactly rationalize how these affect the final rates. On the one hand, multiple conformers of transition structures and the respective reaction fluxes were taken into account in **F**, which will increase the computed rates. On the other hand, the values in **E** were obtained under the microcanonical regime, which is more in line with the experimental conditions by which the rates were derived.

Finally, we have the data-driven predictions in entry **G**. The rate constants are slightly overestimated, with a rather small range. All values were predicted to lie between 1.9 and 4.3 × 10^–11^ cm^3^ s^–1^. Nonetheless, it is interesting to see how this approach fared in comparison to the more conventional quantum chemical protocols. The predictions for the two fastest reactions in the test sets (1 and 5) are in extraordinary agreement with the experimental values. The lower reactivity in the other cases does not appear to be captured by the model. At least for reaction 4, this is likely due to the disregard of steric effects, as no suitable reference data could be included.

### 3.3 Benchmarking

Obviously, the value of any benchmarking study depends on the quality of the benchmark data, i.e., the rate constants *k*_exp_ of gas-phase ion-molecule reactions measured in a QIT in the present case. This methodology is well established and known to be quite reliable.^13,14,17,18^ However, for the explicit purpose of benchmarking, it is worth reviewing possible sources of errors in the experiments.

Statistical errors could be simply derived from the standard deviation of the rate constants determined for different measurements. In most cases, these deviations are relatively small (≤20%, 2σ), reflecting the robustness and good reproducibility of the experiments. The only exception is reaction 4, for which the combination of a sluggish formation of the reactant ion and the fast consumption of the latter resulted in low absolute signal intensities. These low absolute intensities translated into reduced signal-to-noise ratios and thus to large statistical errors (71%).

The estimation of systematic errors is more difficult. Presumably, the most significant error here is the uncertainty in the substrate concentration *N*_substrate_/*V* within the QIT due to the difficulties associated with the accurate determination and control of absolute pressures in the given regime. However, the control experiments performed at different substrate concentrations showed a satisfactory linear correlation with the measured pseudo-firs-order rate constants. As discussed above, this finding lends additional confidence to the determined bimolecular rate constants and suggests that the latter are well within the previously estimated uncertainty limits of ±30%.^20^ Another possible systematic error might arise from the assumption of a temperature of *T* = (310  ±  20) K within the QIT. This value goes back to studies by Gronert^17^ and was later confirmed by O’Hair and coworkers.^18^ Given that the QIT operates at ambient temperature and contains He buffer gas for thermalizing the stored ions, the estimated temperature indeed seems quite plausible. Lastly, errors might arise from the neglect of ions of low abundance (≤5%) in the kinetic analysis. However, tests showed that such minor species do not change the obtained rate constants significantly.

For the actual comparison of the predictions of the quantum chemical calculations with the experimental benchmarks, the former must first be converted into theoretical rate constants (*k*_theo_) by means of statistical-rate theory calculations (except for submissions **G**). Thus, the accuracy of these statistical-rate theory calculations also needs to be critically evaluated. In order to run the kinetic simulations with the MESMER software package, the double-well potential of the gas-phase protolysis reactions had to be simplified by disregarding the final step, i.e., the dissociation of the product complex into separated products. As the proton-transfer step is associated with the highest barrier and thus will be the rate-determining step, this approximation should not be problematic.

A more important question concerns the suitability of the description of the examined system as a microcanonical or a canonical ensemble. As the number of collisions in the gas-phase experiments is relatively low, the energy flow between the individual particles is limited, which suggests that the description of a microcanonical ensemble should be adequate. In accordance with this notion, analogous previous studies also rested on the assumption of a microcanonical ensemble and achieved excellent agreement with the predictions of high-level quantum chemical calculations.^13,14^ Directly comparing two different alternatives in the present work (for the datasets **E** and **F**) does not necessarily solve the questions around the adequacy of the model. In **F**, although a canonical approach is used, there is no assumption of thermal equilibrium with the reactant complexes. One is assuming instead a quasi-equilibrium with the free reactants. **E** itself also has shortcomings when compared to **F**, given that it assumes a single pathway for the reaction. The data provide an interesting basis for discussion, but no definite conclusions are drawn yet. It would be easier to address these questions in zincate systems, where the electronic structure problem is less of an issue.

Finally, the kinetic simulation also involves the calculations of ion-neutral collision rates (for entries **A**–**D**) according to the capture theory by Su and Chesnavich.^22−24^ This theory treats ions as point charges and therefore tends to underestimate the collision rates for large ions, such as the ones probed in the present study.^59^ However, as the reactions analyzed here do not approach the collision-rate limit, the remaining uncertainty in the calculation of the latter should not give rise to a significant error. As the analysis described above demonstrates, the applied benchmarking approach itself is not supposed to introduce considerable uncertainties or systematic errors. Thus, it indeed seems to be apt for gauging the performance of the different quantum theoretical predictions.

**Figure 5:**
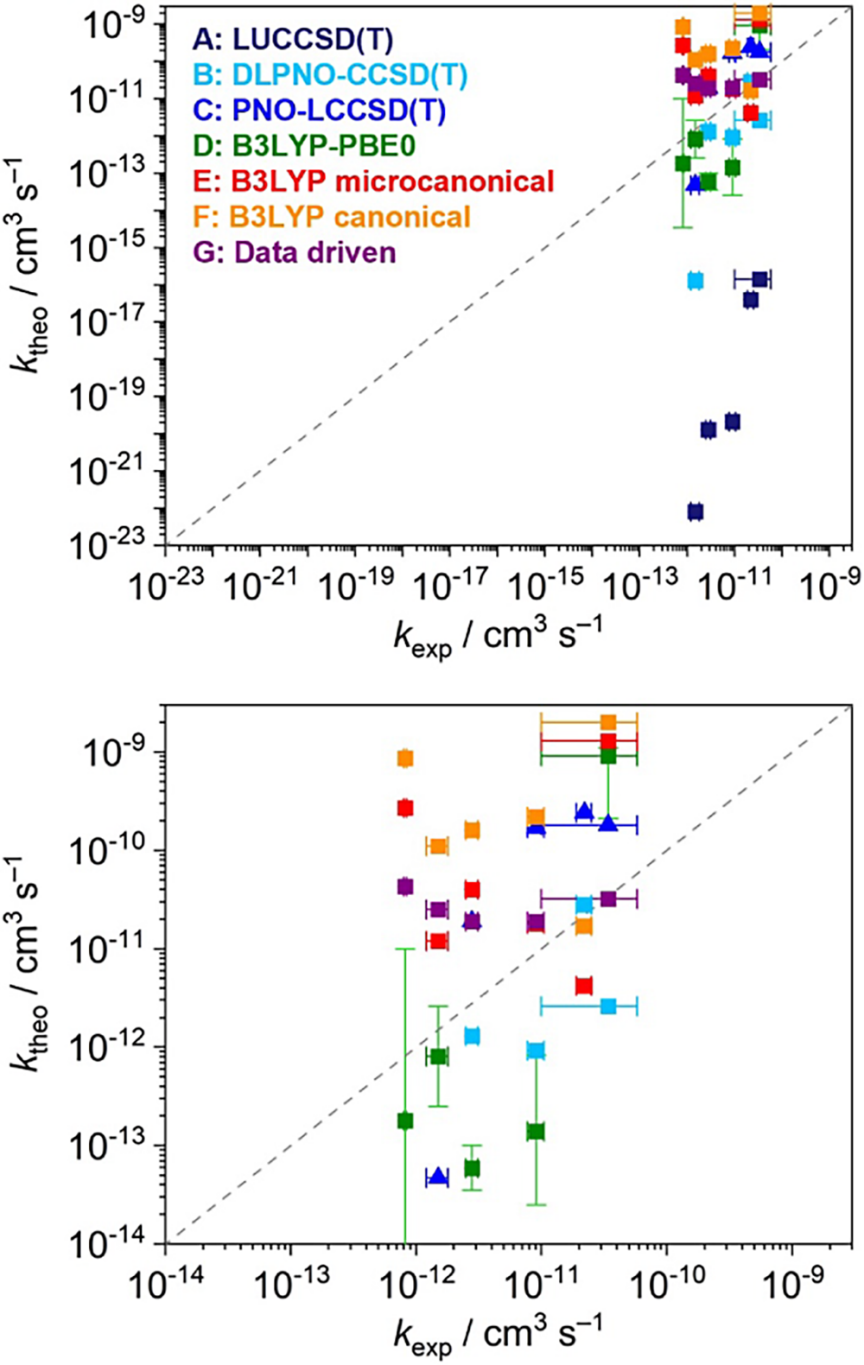
Correlation of the experimental and theoretical rate constants, *k*_exp_ and *k*_theo_, respectively, over the entire range (top) and zoom-in (bottom). The gray dashed line corresponds to ideal agreement between experiment and theory.

Figure 5 (top) shows the correlation for all entries with the measured rate constants, *k*_exp_. Due to the large deviations from entry **A**, this is not very informative. The graphic spans 14 orders of magnitude alone because of the latter values. We focus instead on Figure 5 (bottom), which provides a better scale for the remaining submissions. As previously noted, the experimental rate constants follow the reaction order *k*(4) < *k*(3) < *k*(2) < *k*(1) < *k*(0) < *k*(5) for the test systems. This trend is valid even when considering the experimental uncertainties. The only exception is the ordering between 0 and 5, so we are not taking this ordering into account. Entry **C** is the only dataset that replicates the order if we ignore the fact that reaction 4 was not computed. Entry **B**, which is computationally very similar to **C** (DLPNO-UCCSD(T) instead of PNO-LUCCSD(T)-F12) swaps the order of reactions 1 and 2. All other entries fail because of the ordering of more than one reaction. Overall, as expected, the results from **B** and **C** are rather similar and among the sets that best correlate with the experimental data. A large outlier is observed for set **B** in the case of reaction 3. It is, however, difficult to conclude whether the small differences observed between the two entries are linked to the specific PNO implementation or the use of explicit correlation in **C** (which leads to an improved one-particle space description).

In the case of submission **D**, only 2 out of 5 reactions are within the estimated uncertainty (reactions 1 and 5). This is not a very positive result, showing that the DFT parametrization is not a one-size-fits-all solution. Instead, it demonstrates that both B3LYP-D3(BJ) and PBE0-D3(BJ) cannot cover the full range, even when adjusted for one system. The problems in finding tailored parameterizations in hybrid functionals for Fe complexes were recently highlighted.^60^ The same type of issue appears again in this particular application. Going into further detail, one could also observe whether the estimated theoretical error bar correlates with the deviations from the experiment. This does not seem to be the case, with reaction 2 bearing not only the smallest error bar but also the largest deviation. It would be of interest to investigate whether other combinations of functionals could do a better job.

In the case of **F**, the largest error is observed for reaction 4 (rate constant overestimated by a factor of about 300). All other results are within 2 orders of magnitude, which for DFT is quite reasonable.

Entry **G** roughly estimates that all of the reactions are fairly similar. It even predicts the same rate constant for reactions 1 and 2. This is not in line with the experiment, but it does a good job in roughly predicting the absolute values. The largest deviations are observed for reactions 3 and 4, where this entry predicts an increase rather than a decrease in the rate constant relative to reaction 0, which was used as a reference value in the corresponding prediction workflow. In fact, and quite surprisingly, it is the entry with the lowest maximum ratio (*k*_theo_/*k*_exp_ or *k*_exp_/*k*_theo_, depending on which value is larger). In our comparisons, we mostly consider the ratio between the computed and experimental rates. The largest ratio in entry **G** is found for reaction 4, with a factor of 52. This is likely affected by the model disregarding steric effects due to the absence of suitable reference data that would allow one to model its influence on the reaction rate. Finally, it is worth noting that this approach requires by far the least computational resources.

Many of the entries are likely affected by an incomplete search of the reaction space. With the structures provided in the Supporting Information, other groups are given the opportunity to build multiple reaction pathway models or even search for further, more stable intermediates and transition states.

## 4 Conclusions

In this contribution, we report on experimentally derived rate constants for the protonation of a series of trisarylferrates. These are accompanied by unbiased theoretical predictions from different groups, showcasing a variety of computational approaches.

The protolysis reactions with the proton donors R^F3^OH and R^F2^OH proceeded in a way analogous to that previously observed for trisarylzincate complexes.^13^ For the protonation of FePh_3_^–^ and ZnPh_3_^–^ by R^F3^OH, the determined reaction efficiencies were quite similar (*φ* = 1.8 vs. 1.3%). In contrast, the resulting product ions FePh_2_(OR^F3^)^−^ and ZnPh_2_(OR^F3^)^−^ differed in their behavior in that the former underwent consecutive protolysis reactions, whereas the latter did not to a notable extent. The reason for this deviating reactivity at the stage of the alkoxy-ligated ferrate and zincate complexes is unclear. Raising the electron density of the aryl groups facilitates their protonation, whereas increased steric demands have the opposite effect. Altogether, the experiments furnished a set of 6 rate constants evenly distributed over a range of a factor of 40 and, thus, proved well suited for the benchmarking of theoretical calculations and data-driven predictions.

Based on the experience with other systems,^13^ we deemed microcanonical modeling to be the most adequate way to connect the computed barriers to kinetic rate constants. In the present work, it is difficult to reach an authoritative conclusion. The conformational search should be further extended. This would enable a more balanced comparison.

Another burning issue is electronic structure treatment. The overall quality of the DFT results was lower than that of the local coupled cluster. However, only a small number of functionals was tested. It would be of interest to see how the performance of different kernels aligns with benchmarks carried out on smaller systems. One can also clearly observe that the PNO approaches are much closer to a black-box computational tool in comparison to the older PAO variant. However, the overall robustness of the protocols is still lacking. Each set had at least one significant outlier, either in the absolute sense or in the relation between the different reactions. Some of the points that could be pursued in further computational studies include: basis set dependence of coupled-cluster calculations; impact of multireference on the overall results; search for DFT functionals with robust predictions for both zincates and ferrates; and measures of computational uncertainty for reaction rate constant predictions.

Despite the somewhat disappointing performance of most theoretical methods, this work offers ideal conditions for further computational studies. This is true for both the quantum chemical and the kinetic modeling simulations. It is possible to roughly estimate for each of these reactions a range for the barrier height based on the experimental measurements and under some constraints provided by the theoretical calculations. We are currently exploring the use of Bayesian statistics to combine the two sets of data. These barriers, in turn, can be more directly compared to computations for the reactants and transition state. Other groups can also make use of the submitted data to evaluate different kinetic modeling approaches.

The only submission that was data driven (entry **G**) was able to predict roughly the range of the rate constants but was unable to replicate the relative trends. Nevertheless, compared to the computationally much more demanding submissions based on quantum chemistry, it provided competitive predictions. Also here, one sees room for further improvement, first and foremost by combining more comprehensive data sources, relying on more careful assumptions regarding the transferability of linear free energy relationships, or exploring better descriptors for the mathematical relation.

The present study demonstrates that the current experimental methodology is able to provide absolute bimolecular rate constants as benchmarks for theoretical calculations. Extending such measurements to additional organometallic complexes promises to show how the replacement of the metal center or organyl ligand influences the microscopic reactivity. For the experiment, it makes no difference whether the reactant complex contains an open d-electron shell metal, such as iron, or a closed d-electron shell, such as in the case of zinc. In contrast, the former poses a significantly greater challenge to quantum chemical calculations, as the present study directly shows. Whether data-driven approaches will be able to capture the differences when exchanging metal centers is also a question of great interest. Future efforts should focus on finding theoretical methods for improved performance in describing the reactivity of transition-metal complexes. For this purpose, a larger set of experimental benchmarks will be of key importance.

## Supporting Information Available

1. Reaction schemes, list of participants, reaction networks used to interpret the data, correlation plots for the rate constants and substrate volume, time-dependent intensity profiles and respective fits, ESI data and individual theory results. Computational details to all theory submissions are provided in separate additional files (PDF)
