# Supplementary material for: The Fe-MAN Challenge: Ferrates–Microkinetic Assessment of Numerical Quantum Chemistry
Source: J Phys Chem A. 2024 Jun 4;128(23):4663–73. doi: 10.1021/acs.jpca.4c01361 (PMC11182345; doi:10.1021/acs.jpca.4c01361)
Supplement: Supplementary file 1 — jp4c01361_si_001.pdf [file jp4c01361_si_001.pdf]

Supporting Information (Main)
The Fe-MAN Challenge: Ferrates – Microkinetic Assessment of Numerical Quantum Chemistry

Rene Rahrt[a], Björn Hein-Janke[b], Kosala N. Amarasinghe[c], Muhammad Shafique[c], Milica Feldt[c], Luxuan Guo[d], Jeremy N. Harvey[d], Robert Pollice[e], Konrad Koszinowski[a,]*, Ricardo A. Mata[b,]*

a) Universität Göttingen, Institut für Organische und Biomolekulare Chemie, Tammannstr. 2, Göttingen 37077, Germany.
b) Universität Göttingen, Institut für Physikalische Chemie, Tammannstr. 6, Göttingen 37077, Germany.
c) Leibniz Institute for Catalysis (LIKAT), Albert-Einstein-Str. 29A, 18059 Rostock, Germany
d) Department of Chemistry, KU Leuven, Celestijnenlaan 200F, B-3001 Leuven, Belgium
e) Stratingh Institute for Chemistry, University of Groningen, Nijenborgh 4, 9747 AG Groningen, The Netherlands

Supporting Information

Table of contents

1 Investigated reactions of organoferrate anions with alcohols 2
2 Participants and submission 3
3 Further information about the experimental methods 4
4 Experimental results 6
5 Theoretical results 9

1 Investigated reactions of organoferrate anions with alcohols

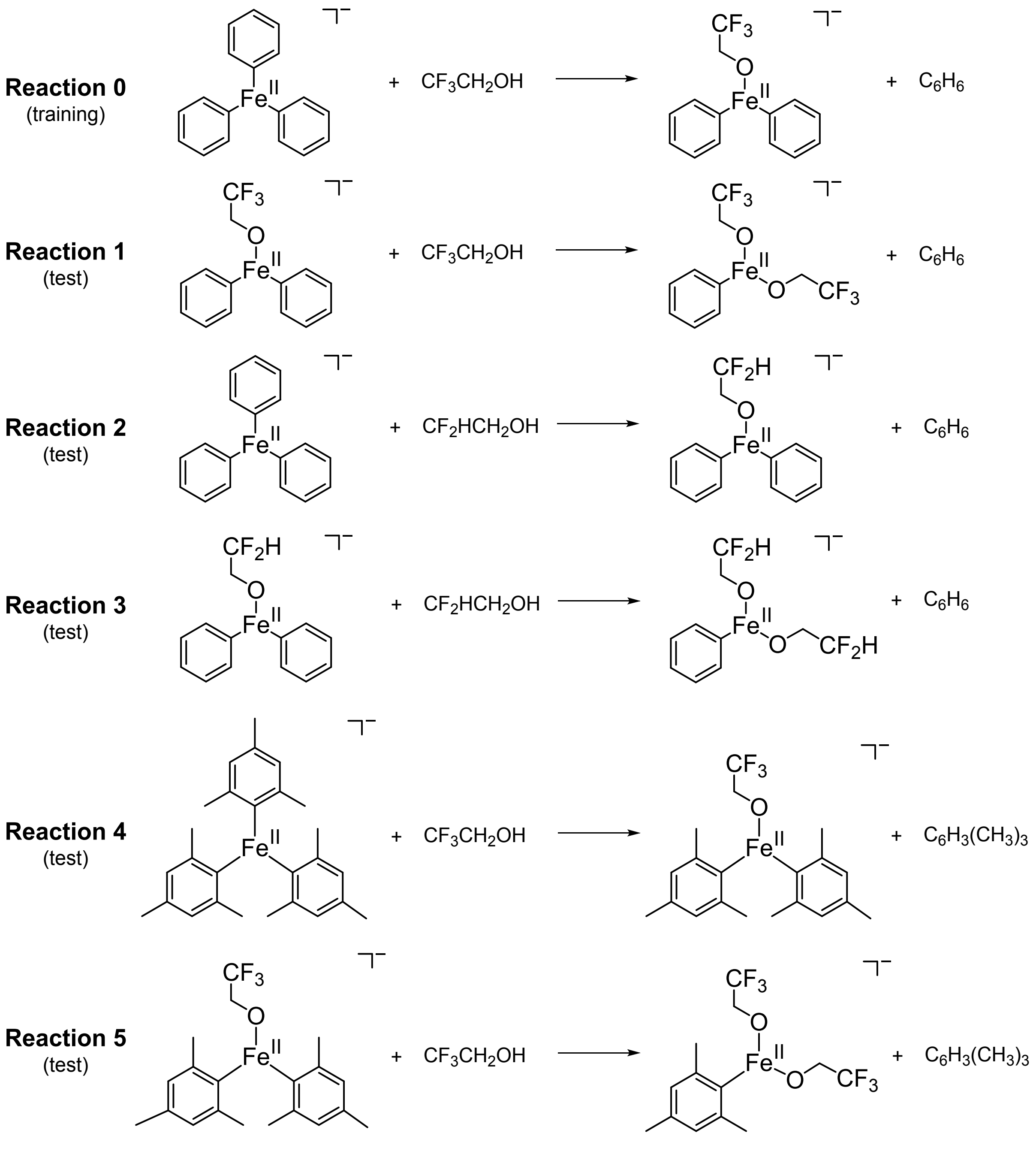

Scheme S1: Investigated protolysis reactions of the organoferrate anions with the proton donors ROH.

2 Participants and submission

Table S1: Overview of the participants and their submissions for the Fe-MAN challenge.

Submission	Structure optimization	Electronic single-point energies	Kinetic model	Participants
**A: LUCCSD(T)**	ωB97X-D3/ def2-TZVP	LUCCSD(T)/ def2-TZVP	microcanonical; Master equation calculations	Amarasinghe, Shafique, Feldt
**B: DLPNO-CCSD(T)**	ωB97X-D3/ def2-TZVP	DLPNO-CCSD(T)/ def2-TZVP	microcanonical; Master equation calculations	Amarasinghe, Shafique, Feldt
**C: PNO-LCCSD(T)**	ωB97X-D3/ def2-TZVP	PNO-LCCSD(T)-F12/def2-TZVP	microcanonical; Master equation calculations	Amarasinghe, Shafique, Feldt
**D: B3LYP-PBE0**	BP86-D3BJ/ def2-SVP	B3LYP-D3BJ+PBE0/ def2-TZVP	microcanonical; Master equation calculations	Hein-Janke, Mata
**E: B3LYP microcanonical**<br>**F: B3LYP canonical**	B3LYP-D3BJ/ def2-SVP	B3LYP-D3BJ/ def2-TZVPD	microcanonical; Master equation calculations<br>canonical, TST	Guo, Harvey
**G: Data driven**	Data-driven model			Pollice

3 Further information about the experimental methods

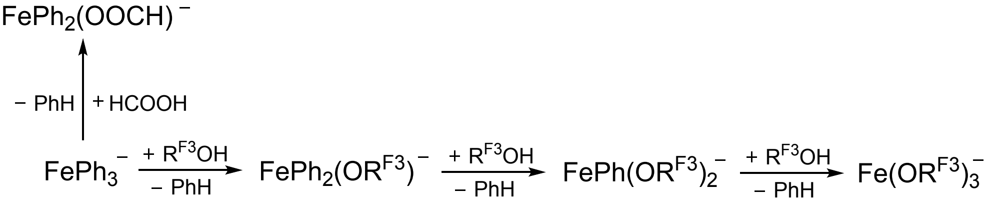

Scheme S2: Reaction network for the protolysis reactions of $FePh_3^-$ by CF_3CH_2OH ($R^{F3}OH$).

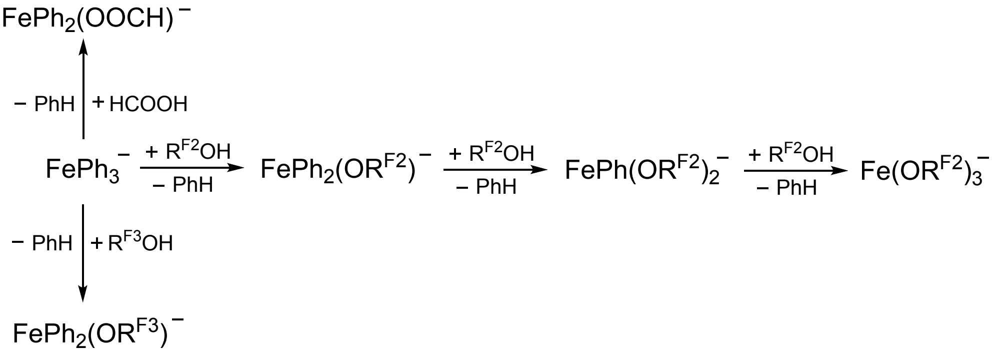

Scheme S3: Reaction network for the protolysis reactions of $FePh_3^-$ by CF_2HCH_2OH ($R^{F2}OH$).

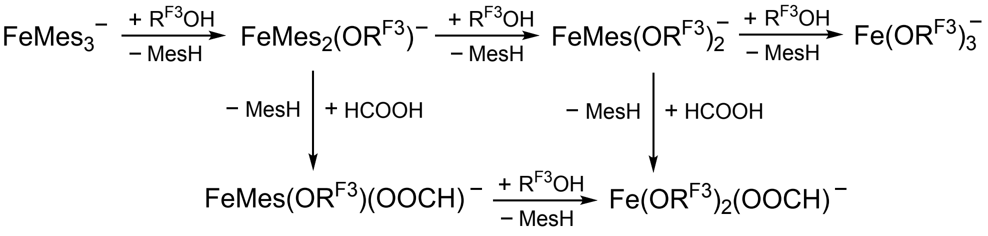

Scheme S4: Reaction network for the protolysis reactions of $FeMes_3^-$ by CF_3CH_2OH (ROH).

Table S2: Theoretical collision rate constants k_{coll} of the reactant ions with the neutral alcohols at T = 310 K according to the capture theory of Su and Chesnavich.

	Reactant ion			
**Reaction #**	**Species[a]**	***m/z***	**Alcohol[b]**	k_{coll} **/ 10^{-9} cm^3 s^{-1}**
0	$FePh_3^-$	287.16	$R^{F3}OH$[c]	1.2228
1	$FePh_2(OR^{F3})^-$	309.08	$R^{F3}OH$[c]	1.2115
2	$FePh_3^-$	287.16	$R^{F2}OH$[d]	0.7602
3	$FePh_2(OR^{F2})^-$	291.09	$R^{F2}OH$[d]	0.7591
4	$FeMes_3^-$	413.40	$R^{F3}OH$[c]	1.1736
5	$FeMes_2(OR^{F3})^-$	393.25	$R^{F3}OH$[c]	1.1794

[a] Ph = C_5H_6, Mes = $C_6H_2(CH_3)_3$. [b] $R^{F3}OH$ = CF_3CH_2OH, $R^{F2}OH$ = CF_2HCH_2OH. [c] For the dipole moment and the polarizability volume, calculated values of $\mu(R^{F3}OH)$ = 1.95 D and $\alpha'(R^{F3}OH)$ = 4.38 Å^3 were used, respectively. [d] For the dipole moment and the polarizability volume, calculated values of $\mu(R^{F2}OH)$ = 0.72 D and $\alpha'(R^{F2}OH)$ = 4.37 Å^3 were used, respectively.

4 Experimental results

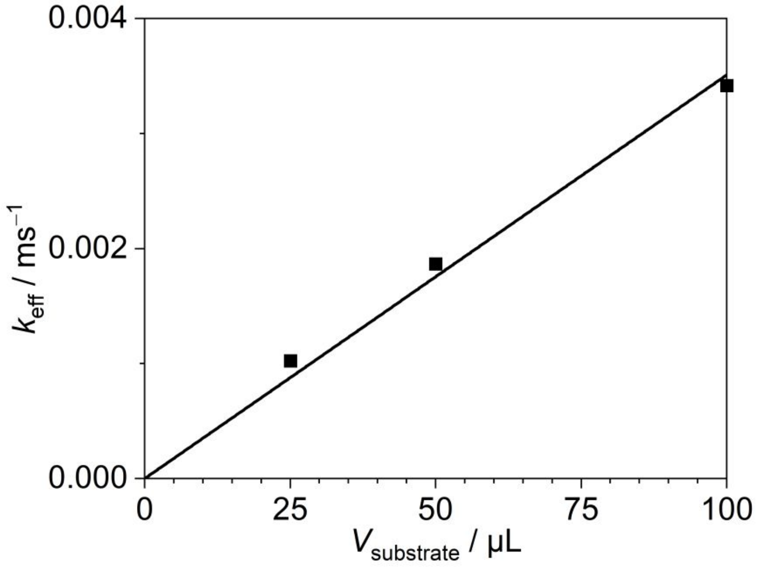

Figure S1: Correlation of the effective rate constant k_{eff} for the protonation of Ph_3Fe^- by CF_3CH_2OH against the introduced volume of the substrate $V_{substrate}$ (black squares) and linear fit with the intercept set to 0 (R^2 = 0.997).

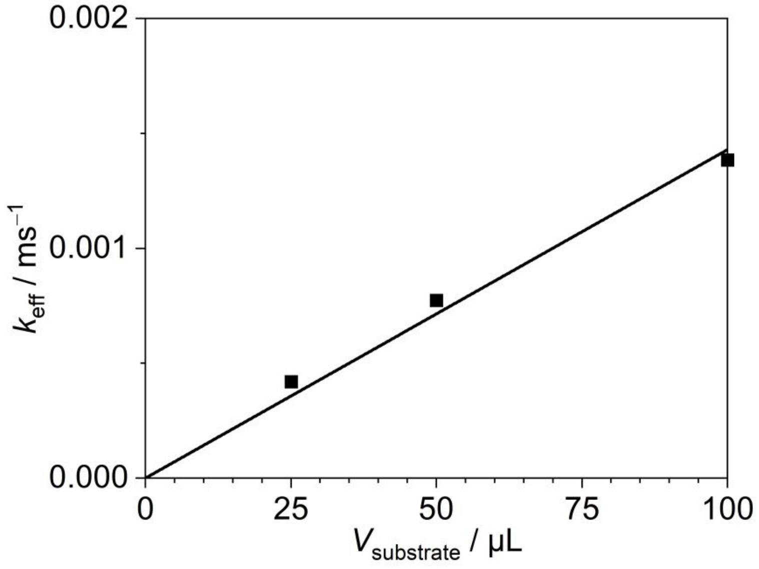

Figure S2: Correlation of the effective rate constant k_{eff} for the protonation of $Ph_2Fe(OR)^-$ by CF_3CH_2OH against the introduced volume of the substrate $V_{substrate}$ (black squares) and linear fit with the intercept set to 0 (R^2 = 0.998).

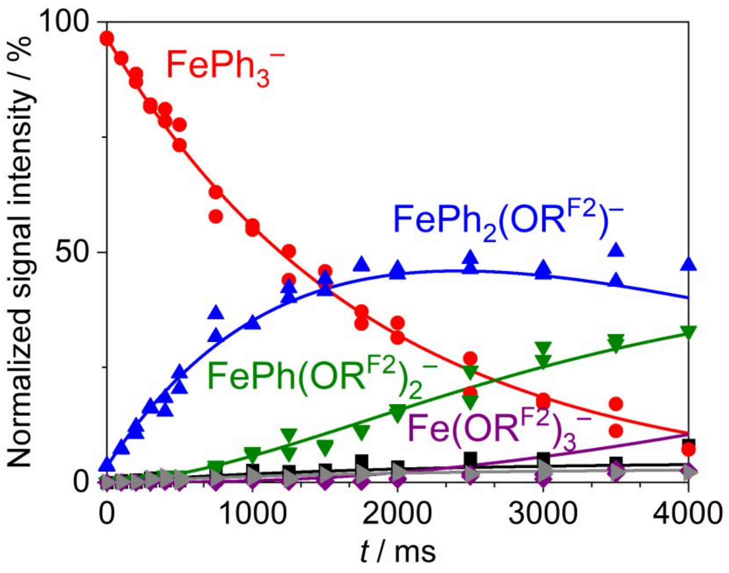

Figure S3: Time-dependent intensity profile (MS^n) for the gas-phase ion-molecule reaction of mass-selected $FePh_3^-$ with 2,2-difluoroethanol (CF_2HCH_2OH; $R^{F2}OH$) and its products as well as fit of the data according to Scheme S3.

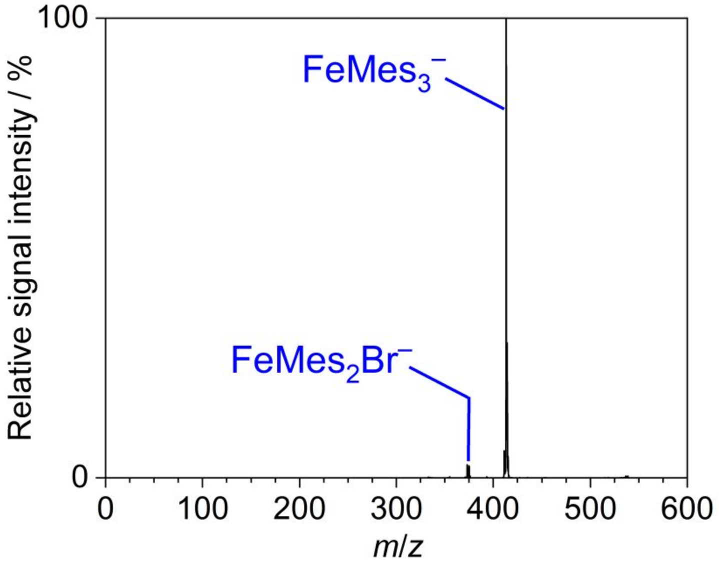

Figure S4: Negative ion-mode electrospray ionization (ESI) mass spectrum (MS^1) of a solution of $Fe(acac)_3$ and MesMgBr (4 equiv) in THF (10 mM) at 195 K.

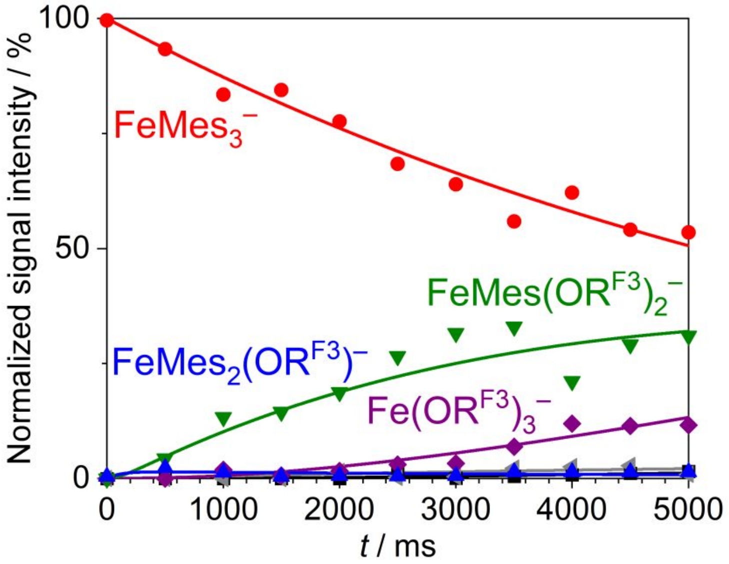

Figure S5: Time-dependent intensity profile (MS^n) for the gas-phase ion-molecule reaction of mass-selected $FeMes_3^-$ with 2,2,2-trifluoroethanol (CF_3CH_2OH; $R^{F3}OH$) and its products as well as fit of the data according to Scheme S4.

5 Theoretical results

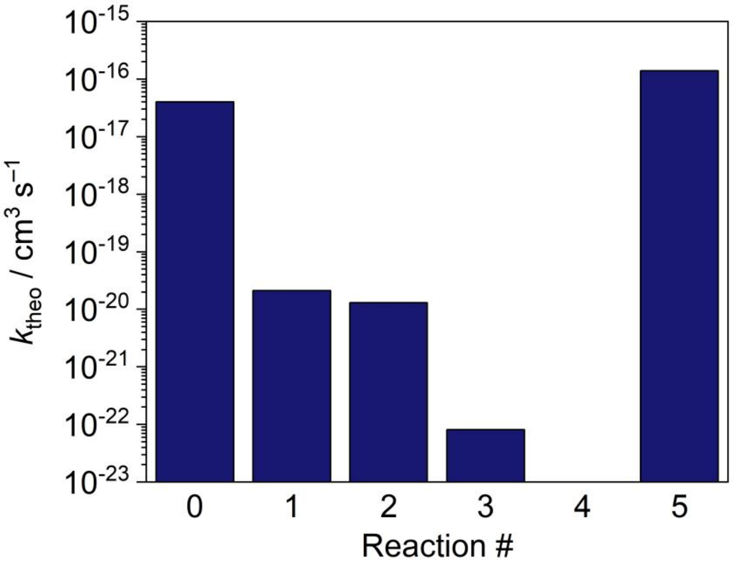

Figure S6: Theoretical rate constants k_{theo} as obtained from Master-equation calculations based on the stationary-point structures and energies which were computed with the quantum-chemical method LUCCSD(T)/def2-TZVP//ωB97X-D3/def2-TZVP.

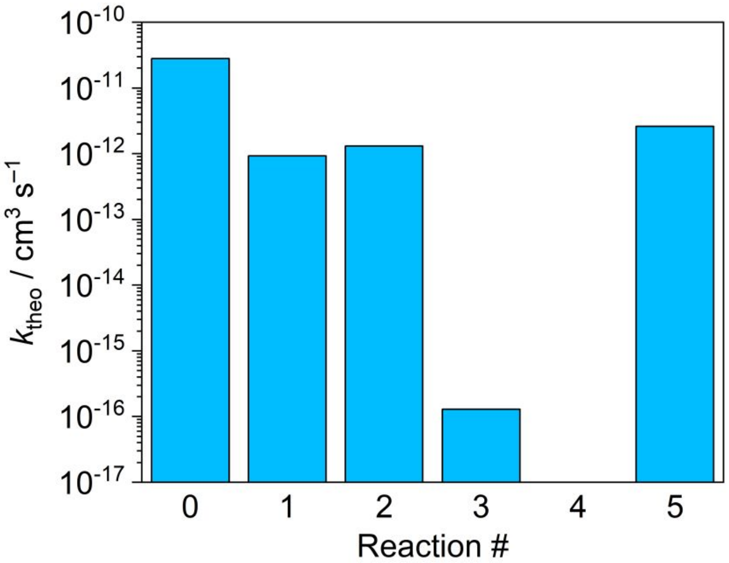

Figure S7: Theoretical rate constants k_{theo} as obtained from Master-equation calculations based on the stationary-point structures and energies which were computed with the quantum-chemical method DLPNO-CCSD(T)/def2-TZVP///ωB97X-D3/def2-TZVP.

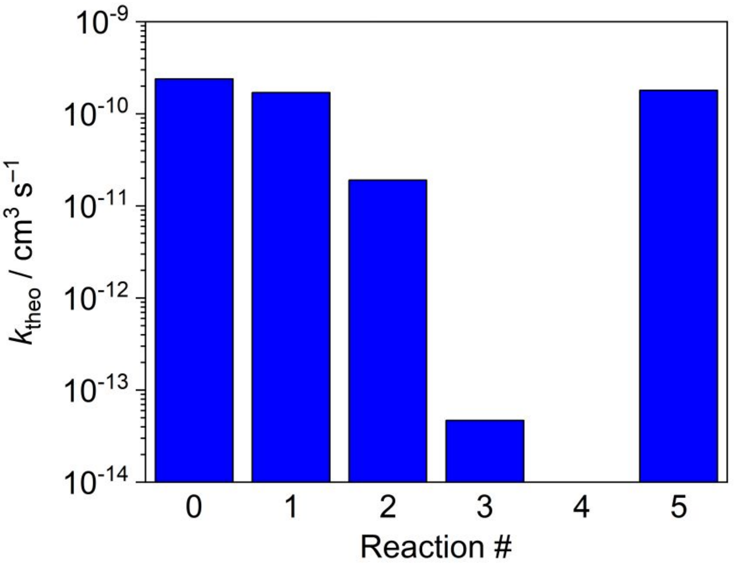

Figure S8: Theoretical rate constants k_{theo} as obtained from Master-equation calculations based on the stationary-point structures and energies which were computed with the quantum-chemical method PNO-LCCSD(T)-F12/def2-TZVP//ωB97X-D3/def2-TZVP.

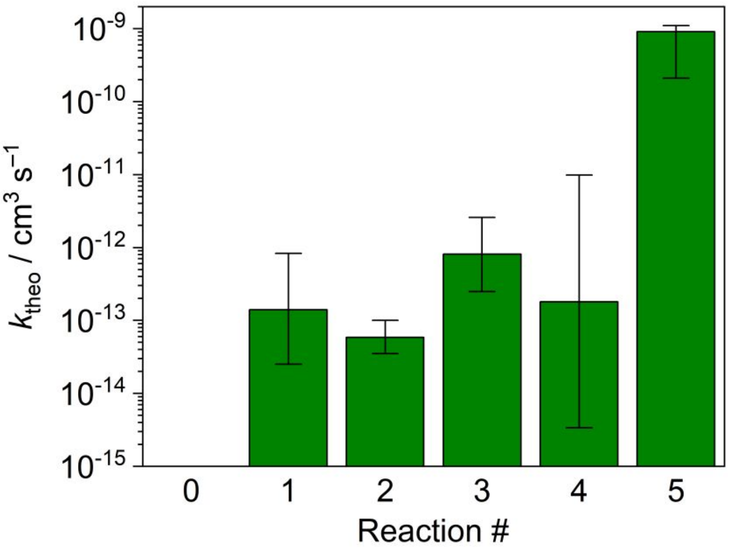

Figure S9: Theoretical rate constants k_{theo} and uncertainties as obtained from Master-equation calculations based on the stationary point structures and energies which were computed employing a combination of the density functionals B3LYP and PBE0 with def2-TZVP basis sets.

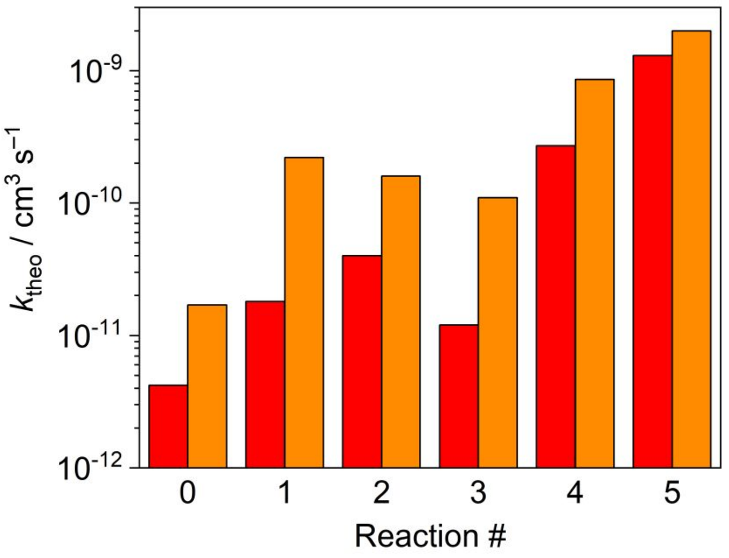

Figure S10: Theoretical rate constants k_{theo} as obtained from microcanonical Master-equation calculations (red) or canonical transition-state theory calculations (orange) based on the stationary-point structures and energies which were computed with the quantum-chemical method B3LYP-D3BJ/def2-TZVPD//B3LYP-D3BJ/def2-SVP.

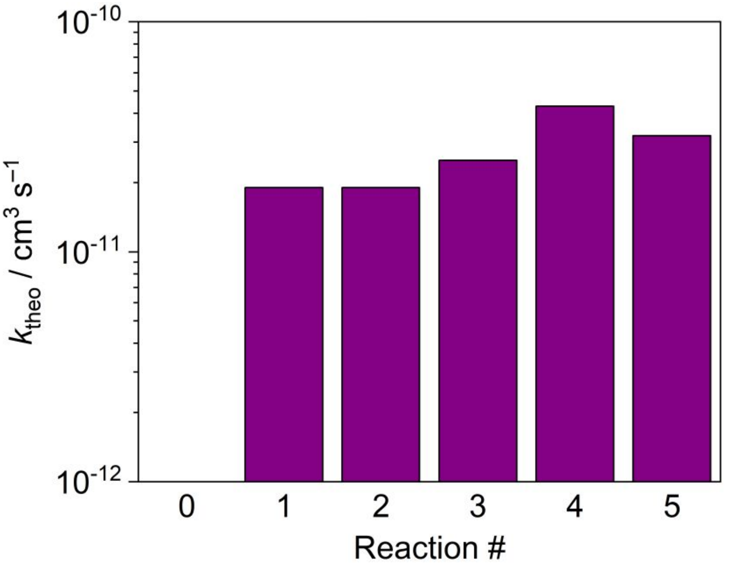

Figure S11: Theoretical rate constants k_{theo} as obtained from the data-driven model.
