# Supplementary material for: The Fe-MAN Challenge: Ferrates–Microkinetic Assessment of Numerical Quantum Chemistry
Source: J Phys Chem A. 2024 Jun 4;128(23):4663–73. doi: 10.1021/acs.jpca.4c01361 (PMC11182345; doi:10.1021/acs.jpca.4c01361)
Supplement: Supplementary file 2 — jp4c01361_si_002.pdf [file jp4c01361_si_002.pdf]

Supporting Information (Entry A)
The Fe-MAN Challenge: Ferrates – Microkinetic Assessment of Numerical Quantum Chemistry

Rene Rahrt[a], Björn Hein-Janke[b], **Kosala N. Amarasinghe[c]**, **Muhammad Shafique[c]**, **Milica Feldt[c]**, Luxuan Guo[d], Jeremy N. Harvey[d], Robert Pollice[e], Konrad Koszinowski[a,*], Ricardo A. Mata[b,*]

a) Universität Göttingen, Institut für Organische und Biomolekulare Chemie, Tammannstr. 2, Göttingen 37077, Germany.
b) Universität Göttingen, Institut für Physikalische Chemie, Tammannstr. 6, Göttingen 37077, Germany.
c) Leibniz Institute for Catalysis (LIKAT), Albert-Einstein-Str. 29A, 18059 Rostock, Germany
d) Department of Chemistry, KU Leuven, Celestijnenlaan 200F, B-3001 Leuven, Belgium
e) Stratingh Institute for Chemistry, University of Groningen, Nijenborgh 4, 9747 AG Groningen, The Netherlands

1 Computational Details

All structures were optimized with the ωB97X-D3 functional and the def2-TZVP basis set. For the convergence the following criteria were used: *VeryTightSCF TightOpt SlowConv*. ZPE corrections were obtained from these calculations. Standard atomic weights were used for the atomic masses for vibrational calculations. All optimization calculations were performed with ORCA 5.0.3.

For all ωB97X-D3/def2-TZVP optimized structures, electronic single-point energy calculations were carried out with LUCCSD(T0) approach in combination with def2-TZVP basis set. Density fitting was applied in all calculations in combination with corresponding basis sets (def2-TZVP/JKFIT and def2-TZVP/MP2FIT). Preliminary DFT calculations (using B-LYP or BP86) were carried out to improve the convergence of the subsequent HF calculations. In all local calculations the intrinsic bond orbital localization was used for orbitals, while orbital domains were determined according to the natural population analysis (NPA) criteria with T_{NPA} = 0.03. The distance criteria was used for the classification of the orbital pairs. Strong pairs were defined within the distance of 3 bohr and close pairs within 5 bohr. Strong orbital pairs were fully included in the coupled cluster part and the MP2 amplitudes of the close pairs were included in the calculations for strong pairs (keepcl=1). All the LUCCSD(T0) calculations were performed with Molpro 2022.1.
