# Supplementary material for: The Fe-MAN Challenge: Ferrates–Microkinetic Assessment of Numerical Quantum Chemistry
Source: J Phys Chem A. 2024 Jun 4;128(23):4663–73. doi: 10.1021/acs.jpca.4c01361 (PMC11182345; doi:10.1021/acs.jpca.4c01361)
Supplement: Supplementary file 3 — jp4c01361_si_003.pdf [file jp4c01361_si_003.pdf]

Supporting Information (Entry B)
The Fe-MAN Challenge: Ferrates – Microkinetic Assessment of Numerical Quantum Chemistry

Rene Rahrt[a], Björn Hein-Janke[b], **Kosala N. Amarasinghe[c]**, **Muhammad Shafique[c]**, **Milica Feldt[c]**, Luxuan Guo[d], Jeremy N. Harvey[d], Robert Pollice[e], Konrad Koszinowski[a,*], Ricardo A. Mata[b,*]

a) Universität Göttingen, Institut für Organische und Biomolekulare Chemie, Tammannstr. 2, Göttingen 37077, Germany.
b) Universität Göttingen, Institut für Physikalische Chemie, Tammannstr. 6, Göttingen 37077, Germany.
c) Leibniz Institute for Catalysis (LIKAT), Albert-Einstein-Str. 29A, 18059 Rostock, Germany
d) Department of Chemistry, KU Leuven, Celestijnenlaan 200F, B-3001 Leuven, Belgium
e) Stratingh Institute for Chemistry, University of Groningen, Nijenborgh 4, 9747 AG Groningen, The Netherlands

1 Computational Details

All structures were optimized with the ωB97X-D3 functional and the def2-TZVP basis set. For the conver gence the following criteria were used: *VeryTightSCF TightOpt SlowConv*. ZPE corrections were obtained from these calculations. Standard atomic weights were used for the atomic masses for vibrational calculations. All optimization calculations were performed with ORCA 5.0.3.

For all ωB97X-D3/def2-TZVP optimized structures, electronic single-point energy calculations were carried out with DLPNO-CCSD(T0) and the def2-TZVP basis set and def2-TZVPP/C auxiliary basis set. The RI JCOSX approximation is used with the def2/J auxiliary basis set. 3s3p electrons of iron were correlated. Furthermore, *TightSCF* and *SlowConv* convergence criteria were used. All the DLPNO-CCSD(T0) calculations (*NormalPNO*) were performed with ORCA5.0.3.
