# Supplementary material for: The Fe-MAN Challenge: Ferrates–Microkinetic Assessment of Numerical Quantum Chemistry
Source: J Phys Chem A. 2024 Jun 4;128(23):4663–73. doi: 10.1021/acs.jpca.4c01361 (PMC11182345; doi:10.1021/acs.jpca.4c01361)
Supplement: Supplementary file 4 — jp4c01361_si_004.pdf [file jp4c01361_si_004.pdf]

Supporting Information (Entry C)
The Fe-MAN Challenge: Ferrates – Microkinetic Assessment of Numerical Quantum Chemistry

Rene Rahrt[a], Björn Hein-Janke[b], **Kosala N. Amarasinghe**[c], **Muhammad Shafique**[c], **Milica Feldt**[c], Luxuan Guo[d], Jeremy N. Harvey[d], Robert Pollice[e], Konrad Koszinowski[a,*], Ricardo A. Mata[b,*]

a) Universität Göttingen, Institut für Organische und Biomolekulare Chemie, Tammannstr. 2, Göttingen 37077, Germany.
b) Universität Göttingen, Institut für Physikalische Chemie, Tammannstr. 6, Göttingen 37077, Germany.
c) Leibniz Institute for Catalysis (LIKAT), Albert-Einstein-Str. 29A, 18059 Rostock, Germany
d) Department of Chemistry, KU Leuven, Celestijnenlaan 200F, B-3001 Leuven, Belgium
e) Stratingh Institute for Chemistry, University of Groningen, Nijenborgh 4, 9747 AG Groningen, The Netherlands

1 Computational Details

All structures were optimized with the *ω*B97X-D3 functional and the def2-TZVP basis set. For the convergence the following criteria were used: *VeryTightSCF TightOpt SlowConv*. ZPE corrections were obtained from these calculations. Standard atomic weights were used for the atomic masses for vibrational calculations. All optimization calculations were performed with ORCA 5.0.3.

For all *ω*B97X-D3/def2-TZVP optimized structures, electronic single-point energy calculations were carried out with PNO-LCCSD(T)-F12 (pair natural orbitals) with the def2-TZVP basis set and default parameters. Preliminary DFT calculations (using B-LYP or BP86) were carried out to improve the convergence of the subsequent HF calculations. Density fitting was applied in DFT and HF calculations in combination with corresponding basis sets (def2-TZVP/JKFIT). The CABS singles correction was added to the reference energy. All the PNO-LCCSD(T)-F12 calculations were performed with Molpro 2022.1.
