# Supplementary material for: The Fe-MAN Challenge: Ferrates–Microkinetic Assessment of Numerical Quantum Chemistry
Source: J Phys Chem A. 2024 Jun 4;128(23):4663–73. doi: 10.1021/acs.jpca.4c01361 (PMC11182345; doi:10.1021/acs.jpca.4c01361)
Supplement: Supplementary file 5 — jp4c01361_si_005.pdf [file jp4c01361_si_005.pdf]

Supporting Information (Entry D)
The Fe-MAN Challenge: Ferrates – Microkinetic Assessment of Numerical Quantum Chemistry

Rene Rahrt[a], **Björn Hein-Janke[b]**, Kosala N. Amarasinghe[c], Muhammad Shafique[c], Milica Feldt[c], Luxuan Guo[d], Jeremy N. Harvey[d], Robert Pollice[e], Konrad Koszinowski[a,*], **Ricardo A. Mata[b,*]**

a) Universität Göttingen, Institut für Organische und Biomolekulare Chemie, Tammannstr. 2, Göttingen 37077, Germany.
b) Universität Göttingen, Institut für Physikalische Chemie, Tammannstr. 6, Göttingen 37077, Germany.
c) Leibniz Institute for Catalysis (LIKAT), Albert-Einstein-Str. 29A, 18059 Rostock, Germany
d) Department of Chemistry, KU Leuven, Celestijnenlaan 200F, B-3001 Leuven, Belgium
e) Stratingh Institute for Chemistry, University of Groningen, Nijenborgh 4, 9747 AG Groningen, The Netherlands

1 Computational Details

1.1 General Information

Structure inspection and initial guesses for structure optimizations were done via Avogadro, version 1.2.0 [Avogadro: an open-source molecular builder and visualization tool. Version 1.2.0. http://avogadro.cc]. All structure optimizations, frequency and single point calculations, as well as transition state (TS) searches were performed using the ORCA ab initio package, version 4.2 [https://doi.org/10.1063/5.0004608]. All ORCA calculations were performed using the following alterations to its scf procedure:

```
%scf
MaxIter 5000
DIISMaxEq 5
directresetfreq 1
end
```

Transition state guesses were generated via NEB calculations, using the CRI-NEB implementation currently under development at this research group, the latest version of which can be found at gitlab [https://gitlab.gwdg.de/ccbc-group/neb-optimizer.git]. This implementation used ORCA for energy gradient calculations. Frequency calculations used ORCA's Mass2016 package for atomic masses, which consists of the monoisotopic masses of the most abundant or most stable isotope for each element, as of 2016. The scaling factor used was 1.

1.2 Structure and Frequency calculations

Structures for the reactants, pre-reactive complex, product complex, and the products of all five reactions and the training system were obtained through structure optimizations at the BP86 level [https://doi.org/10.1103/PhysRevA.38.3098] with D3BJ corrections and RI approximation, using def2-SVP with def2/J base sets. The integration grid used was a 770-point Lebedev grid. With this same method, vibrational calculations were performed on the optimized structures, and from the frequency data, the zero

point vibrational energies were computed using ORCA's inbuilt thermochemistry functionality.

Structures of the transition states for the five reactions plus training system were obtained by first performing an NEB-CI calculation. After NEB convergence, the highest energy image of the NEB path was extracted and used as a starting guess for a transition state search using ORCA. After convergence, frequency calculations were performed on the optimized transition state structures, as well zero point vibrational energy calculations, in complete analogy to the non-transition state structures mentioned earlier. The method used for all of these calculations was also BP86-D3BJ with RI approximation, with def2-SVP and def2/J base sets, and a 770-point Lebedev grid.

1.3 Single Point Calculations

Single point energies (SPEs) were calculated from two different methods: B3LYP-D3BJ with def2-TZVP base set, and PBE0 [https://doi.org/10.1103/PhysRevLett.77.3865] with def2-TZVP base set. Both methods were used in conjunction with a 770-point Lebedev grid. First, a parameter fitting was performed on both methods, using the known barrier height of the training system as the fitting target. For both methods, the Hartree-Fock exchange was fitted such that for the training system, the barrier calculated from each method would match the training system's barrier of 30+-4 kJ/mol. In the case of B3LYP, the optimal value found for the Hartree-Fock exchange was 45%, in the case of PBE0, it was 30%. Any mention of B3LYP and PBE0 henceforth refers to these parameter-fitted versions of the two methods. The single point energies of the reactants, pre-reactive complex, transition state, product complex, and products of all five reactions were then calculated with both fitted methods each.

1.4 Enthalpy calculations

The enthalpies at 0 K of all structures were calculated as the sum of the zero point vibrational energies obtained from BP86, and the single point energies obtained from B3LYP and PBE0. This yielded two different enthalpies for each structure, one from B3LYP and one from PBE0.

The submitted enthalpies were calculated from the two sets of enthalpy values as follows: All enthalpy values were calculated as relative to the corresponding reaction's starting point, calculated with the same method. The starting point refers to the sum of the two reactant structures. This yielded relative enthalpies for the four latter stages of each reaction: pre-reactive complex, TS, product complex, as well as the sum of the enthalpies of the two individual products. These relative enthalpies could be meaningfully compared between the two methods. The submitted enthalpy values were then calculated as the arithmetic mean of the two relative enthalpies calculated with the two different methods.

2 Additional computed data

This section contains the relative enthalpy values from the individual methods, the energy profile of each

individual reaction, and a statistical analysis of the results to produce and error estimate. In all tables of this section, 'Pre-RC' refers to the pre-reactive complex, 'TS' to the transition state, 'Post-RC' to the product complex, and 'Products' to the sum of the two product molecules.

2.1 Enthalpy values from individual methods

Table S1D: Relative enthalpy values for each reaction for each method.

	Pre-RC [Eh]	TS [Eh]	Post-RC [Eh]	Products [Eh]
rxn1_b3lyp	-0.0139735	-0.0042554	-0.0533686	-0.0556095
rxn1_pbe0	-0.0128761	-0.0075637	-0.0446200	-0.0518535
rxn2_b3lyp	-0.0138037	-0.0050939	-0.0380223	-0.0530142
rxn2_pbe0	-0.0151998	-0.0050303	-0.0385668	-0.0500069
rxn3_b3lyp	-0.0127904	-0.0027887	-0.0272107	-0.0608865
rxn3_pbe0	-0.0106958	-0.0027340	-0.0297280	-0.0542438
rxn4_b3lyp	-0.0133174	-0.0084741	-0.0761812	-0.0601949
rxn4_pbe0	-0.0030129	-0.0003366	-0.0606835	-0.0571207
rxn5_b3lyp	-0.0228614	-0.0151179	-0.0749789	-0.0641003
rxn5_pbe0	-0.0152305	-0.0108033	-0.0564562	-0.0577901

2.2 Analysis of the individual reactions

This subsection contains the energy profiles of the individual reactions, relative to their starting point, as well as an estimate of the margin of error of each reaction stage, for a 95% confidence interval. The calculation of the error estimate is covered in more detail in subsection 2.3. The starting point of each reaction is not listed, as it is defined as the zero point - it lies always at exactly zero with no error. 'RMSD' refers to root mean square deviation, which is identical to the standard deviation.

Table S2D: Energy profile of reaction 1, with statistical analysis

	Pre-RC [Eh]	TS [Eh]	Post-RC [Eh]	Products [Eh]
Avg. Rel. Energy [Eh]	-0.0134248	-0.0059095	-0.0489943	-0.0537315
RMSD [Eh]	0.0005487	0.0016542	0.0043743	0.0018780
Avg. RMSD [Eh]	0.0021138	0.0021138	0.0021138	0.0021138
95% Conf. Interval [Eh]	0.0017234	0.0017234	0.0017234	0.0017234

Table S3D: Energy profile of reaction 2, with statistical analysis.

	Pre-RC [Eh]	TS [Eh]	Post-RC [Eh]	Products [Eh]
Avg. Rel. Energy [Eh]	-0.0145018	-0.0050621	-0.0382945	-0.0515106
RMSD [Eh]	0.0006980	0.0000318	0.0002723	0.0015036
Avg. RMSD [Eh]	0.0006264	0.0006264	0.0006264	0.0006264
95% Conf. Interval [Eh]	0.0005107	0.0005107	0.0005107	0.0005107

Table S4D: Energy profile of reaction 3, with statistical analysis.

	Pre-RC [Eh]	TS [Eh]	Post-RC [Eh]	Products [Eh]
Avg. Rel. Energy [Eh]	-0.0117431	-0.0027613	-0.0284693	-0.0575652
RMSD [Eh]	0.0010473	0.0000273	0.0012586	0.0033213
Avg. RMSD [Eh]	0.0014136	0.0014136	0.0014136	0.0014136
95% Conf. Interval [Eh]	0.0011525	0.0011525	0.0011525	0.0011525

Table S5D: Energy profile of reaction 4, with statistical analysis.

	Pre-RC [Eh]	TS [Eh]	Post-RC [Eh]	Products [Eh]
Avg. Rel. Energy [Eh]	-0.0081651	-0.0044053	-0.0684323	-0.0586578
RMSD [Eh]	0.0051523	0.0040688	0.0077488	0.0015371
Avg. RMSD [Eh]	0.0046267	0.0046267	0.0046267	0.0046267
95% Conf. Interval [Eh]	0.0037722	0.0037722	0.0037722	0.0037722

Table S6D: Energy profile of reaction 5, with statistical analysis.

	Pre-RC [Eh]	TS [Eh]	Post-RC [Eh]	Products [Eh]
Avg. Rel. Energy [Eh]	-0.0190460	-0.0129606	-0.0657176	-0.0609452
RMSD [Eh]	0.0038155	0.0021573	0.0092613	0.0031551
Avg. RMSD [Eh]	0.0045973	0.0045973	0.0045973	0.0045973
95% Conf. Interval [Eh]	0.0037482	0.0037482	0.0037482	0.0037482

2.3 Error estimation

Since the enthalpy values were computed from two samples (see subsection 1.4), it is possible to perform a basic statistical analysis to estimate the margins of error for the values. The two results from the two methods can be considered like two samples of a measurement, which scatter around the true value with a given variance, which can be estimated from the spread of the sample values, and provide an estimate of the margin of error of the measurements, which in this case are the various enthalpy values.
Doing this for each reaction stage of each reaction individually is problematic, however. In that case, the enthalpy value is computed from a sample size of only 2, which increases the likelihood of statistical flukes distorting the error estimate. For instance, for reactions 2 and 3, the sample values for the TS happen to be very close to one another, resulting in unrealistically small error estimates.

However, when comparing Tables S2D-6D, it becomes apparent that the RMSDs of the individual enthalpy values are similar in size between the stages of a given reaction, while they can differ greatly between reactions. This indicates that it is a reasonable approximation to assume that the sample values for the reaction stages of one reaction all have the same standard deviation. Under this approximation, this shared standard deviation can be computed from the deviations from the mean of all samples, even though the mean differs between samples. It is denoted as 'Avg. RMSD' in Tables S2D-6D.

This shared RMSD is now being calculated with an effective sample size of 8. Now, a reasonable estimate for the margin of error can be calculated, which is shared between all values of a given reaction. The formula for calculating it is

$$\mathrm{MOE} = X \pm t_{8,\,0.95} \cdot {}^{\sigma}/_{\sqrt{n}} \qquad \text{(Eq. 1)}$$

where MOE is the margin of error, σ is the standard deviation, n the sample size (in this case 8), and $t_{8,\,0.95}$ is the student-t-factor for 8 samples and a two-sided 95% confidence interval, which is equal to 2.306. This yields the 95% confidence intervals listed in Tables S2D-6D.
