# Supplementary material for: The Fe-MAN Challenge: Ferrates–Microkinetic Assessment of Numerical Quantum Chemistry
Source: J Phys Chem A. 2024 Jun 4;128(23):4663–73. doi: 10.1021/acs.jpca.4c01361 (PMC11182345; doi:10.1021/acs.jpca.4c01361)
Supplement: Supplementary file 6 — jp4c01361_si_006.pdf [file jp4c01361_si_006.pdf]

Supporting Information (Entries E and F)
The Fe-MAN Challenge: Ferrates – Microkinetic Assessment of Numerical Quantum Chemistry

Rene Rahrt[a], Björn Hein-Janke[b], Kosala N. Amarasinghe[c], Muhammad Shafique[c], Milica Feldt[c], **Luxuan Guo[d]**, **Jeremy N. Harvey[d]**, Robert Pollice[e], Konrad Koszinowski[a,*], Ricardo A. Mata[b]

a) Universität Göttingen, Institut für Organische und Biomolekulare Chemie, Tammannstr. 2, Göttingen 37077, Germany.
b) Universität Göttingen, Institut für Physikalische Chemie, Tammannstr. 6, Göttingen 37077, Germany.
c) Leibniz Institute for Catalysis (LIKAT), Albert-Einstein-Str. 29A, 18059 Rostock, Germany
d) Department of Chemistry, KU Leuven, Celestijnenlaan 200F, B-3001 Leuven, Belgium
e) Stratingh Institute for Chemistry, University of Groningen, Nijenborgh 4, 9747 AG Groningen, The Netherlands

1 Computational Details

All structures were optimized using the B3LYP density functional as implemented in Gaussian 16 rev. A03, using the Grimme dispersion correction with Becke-Johnson damping (we refer to this as B3LYP-D3BJ). For geometry optimization and frequency calculation, we used the def2-SVP basis on all atoms. Vibrational frequencies were computed at the same level of theory. Standard DFT integration grids were used throughout. Refined energies were obtained at the same B3LYP-D3BJ level of theory, but with a larger basis set. Most atoms were described using the def2-TZVPD basis set, but the iron atom was described using the def2-QZVP basis, and all atoms connected to it (i.e. the three C atoms of the Ar_3Fe^- reactants or the two C atoms and one O atom of the $ROFeAr_2^-$ reactants) as well as the O and H atoms of the OH group of the proton donor coreactant were described with the def2-QZVPD basis set [Basis Set Exchange https://www.basissetexchange.org/, DOI: 10.1021/acs.jcim.9b00725]. Standard atomic masses in Gaussian were used for frequency and rotational constant calculations.

The rigid rotor approximation was used for calculating rotational partition functions. For the vibrational partition function, the quasi-harmonic approach [Truhlar et al., DOI: 10.1021/jp205508z] was used. In this approach, spurious imaginary frequencies or real imaginary frequencies smaller in magnitude than 50 cm^{-1} were replaced by real imaginary frequencies of 50 cm^{-1} prior to computing the vibrational partition functions. For the translational partitions functions, a molar volume of 1 cm^3 was used, in accordance with the computation of rate constants in $cm^3\ s^{-1}$.

Care was taken to locate the lowest-energy conformers of all reactants (the alcohol and the anion) **1** as well as of **TS 2-3**. Thereby multiple conformers were obtained for each of them. The contribution of all TSs was taken into account for calculating the overall rate constant, but only the lowest-energy conformer of the reactants was used. The indicated total energies and XYZ files correspond to the lowest-energy conformer for both reactants and TSs.
