# Supplementary material for: The Fe-MAN Challenge: Ferrates–Microkinetic Assessment of Numerical Quantum Chemistry
Source: J Phys Chem A. 2024 Jun 4;128(23):4663–73. doi: 10.1021/acs.jpca.4c01361 (PMC11182345; doi:10.1021/acs.jpca.4c01361)
Supplement: Supplementary file 7 — jp4c01361_si_007.pdf [file jp4c01361_si_007.pdf]

Supporting Information (Entry G)
The Fe-MAN Challenge: Ferrates – Microkinetic Assessment of Numerical Quantum Chemistry

Rene Rahrt[a], Björn Hein-Janke[b], Kosala N. Amarasinghe[c], Muhammad Shafique[c], Milica Feldt[c], Luxuan Guo[d], Jeremy N. Harvey[d], **Robert Pollice[e]**, Konrad Koszinowski[a,*], Ricardo A. Mata[b,*]

a) Universität Göttingen, Institut für Organische und Biomolekulare Chemie, Tammannstr. 2, Göttingen 37077, Germany.
b) Universität Göttingen, Institut für Physikalische Chemie, Tammannstr. 6, Göttingen 37077, Germany.
c) Leibniz Institute for Catalysis (LIKAT), Albert-Einstein-Str. 29A, 18059 Rostock, Germany
d) Department of Chemistry, KU Leuven, Celestijnenlaan 200F, B-3001 Leuven, Belgium
e) Stratingh Institute for Chemistry, University of Groningen, Nijenborgh 4, 9747 AG Groningen, The Netherlands

1 Computational Details

I followed a data-driven approach to predict the protolysis rate constants (k) of organoferrate(II) anions at 310 K. In addition to the single data point for an organoferrate(II) anion provided explicitly for training and data points for protolysis rate constants of organozinc(II) anions published in the literature that were sugge- sted to be used for training [DOI:10.1021/acs.jpca.1c08964], I also used data for protolysis rate constants of organomagnesium(II) anions published previously [DOI:10.1021/ja048476w]. Due to the sparsity of available data for comparable reactions, more sophisticated machine learning approaches could not be employed and I used a classical linear free energy relationship approach. Variation in the observed protolysis rate constant was assumed to stem from one of the following sources: identity of the metal atom (M), the ligands bounds to the metal (L_1, L_2, L_3), the proton donor (D), and the proton-accepting ligand (A). All influences were assumed to stem from electronic factors, thus, steric effects were disregarded as no suitable data to model that influ- ence could be found. Additionally, as only one data point for an organoferrate(II) anion was available, models developed for the various influences on data from other metal complexes were assumed to be directly transferable to iron complexes without further adjustments. Furthermore, all separate influences were assumed to be additive. Potential temperature effects on the various influences considered were disregarded. Table S1G summarizes all the data points used for model building. The following sections provide all details.

1.1 Influence of Ligand

For modeling the influence of the ligand, I only used data points for zinc complexes that had the same acceptor group, i.e. the phenyl group, to disentangle the influence of the ligand from the influence of the acceptor. I used the Hammett equation to model the electronic influence of the ligands and I assumed that the average of the Hammett substituent constants of the ligands would provide an estimate of the overall influence of all the ligands combined. Ligand substituent constants were taken from the literature [DOI:10.1021/cr00002a004]. Additionally, to include the data point with three phenyl substituents in a consistent matter, I corrected the corresponding protolysis rate constant for the entropic advantage of having three equal groups that could all act as acceptors by multiplying the corresponding value by 2/3. Table S2G summarizes the data used to model the influence of the ligand. A moderate linear correlation of the natural logarithm of the rate constant k against the average of the ligand substituent constant ($\sigma_{mean}(L_i)$) resulted in a slope of −14.0 ± 4.4 with an R^2 value of 0.77. This slope was used to model the influence of the ligand.

Table S1G: Data points used for training.

M	L_1	L_2	L_3	D	A	k $[cm^3 s^{-1}]$	Source
Fe	Ph	Ph	Ph	CF_3CH_2OH	Ph	2.20E-11	FeMAN challenge
Zn	Ph	Ph	Ph	CF_3CH_2OH	Ph	1.63E-11	DOI:10.1021/acs.jpca.1c08964
Zn	Ph	Ph	4-OMe-Ph	CF_3CH_2OH	Ph	9.82E-12	DOI:10.1021/acs.jpca.1c08964
Zn	Ph	Ph	4-Me-Ph	CF_3CH_2OH	Ph	1.27E-11	DOI:10.1021/acs.jpca.1c08964
Zn	Ph	Ph	4-F-Ph	CF_3CH_2OH	Ph	6.41E-12	DOI:10.1021/acs.jpca.1c08964
Zn	Ph	Ph	4-Cl-Ph	CF_3CH_2OH	Ph	4.62E-12	DOI:10.1021/acs.jpca.1c08964
Zn	Ph	Ph	4-OMe-Ph	CF_3CH_2OH	4-OMe-Ph	9.16E-12	DOI:10.1021/acs.jpca.1c08964
Zn	Ph	Ph	4-Me-Ph	CF_3CH_2OH	4-Me-Ph	8.86E-12	DOI:10.1021/acs.jpca.1c08964
Zn	Ph	Ph	4-F-Ph	CF_3CH_2OH	4-F-Ph	2.67E-12	DOI:10.1021/acs.jpca.1c08964
Zn	Ph	Ph	4-Cl-Ph	CF_3CH_2OH	4-Cl-Ph	1.39E-12	DOI:10.1021/acs.jpca.1c08964
Mg	CH_3	CH_3COO	CH_3COO	H_2O	CH_3	1.20E-10	DOI:10.1021/ja048476w
Mg	CH_3	CH_3COO	CH_3COO	CH_3OH	CH_3	1.31E-10	DOI:10.1021/ja048476w
Mg	CH_3	CH_3COO	CH_3COO	EtOH	CH_3	1.47E-10	DOI:10.1021/ja048476w

Table S2G: Data points used to model the ligand influence.

M	L_1	L_2	L_3	D	A	k $[cm^3 s^{-1}]$	Source	$\sigma_{mean}(L_i)$	Source
Zn	Ph	Ph	Ph	CF_3CH_2OH	Ph	1.09E-11	Statistical Correction	-0.01	DOI:10.1021/cr00002a004
Zn	Ph	Ph	4-OMe-Ph	CF_3CH_2OH	Ph	9.82E-12	DOI:10.1021/acs.jpca.1c08964	-0.03	DOI:10.1021/cr00002a004
Zn	Ph	Ph	4-Me-Ph	CF_3CH_2OH	Ph	1.27E-11	DOI:10.1021/acs.jpca.1c08964	-0.02	DOI:10.1021/cr00002a004
Zn	Ph	Ph	4-F-Ph	CF_3CH_2OH	Ph	6.41E-12	DOI:10.1021/acs.jpca.1c08964	0.01	DOI:10.1021/cr00002a004
Zn	Ph	Ph	4-Cl-Ph	CF_3CH_2OH	Ph	4.62E-12	DOI:10.1021/acs.jpca.1c08964	0.03	DOI:10.1021/cr00002a004

For the alkoxide groups present in the test set, I derived Hammett substituent constants via a different linear free energy relationship as I could not find the corresponding experimental values. I found that gas-phase acidities of the corresponding alcohols were a good parameter for linear correlation with the corresponding substituent constants of the alkoxides. However, as neither consistent computational nor experimental data was available for all gas-phase acidities, I used a combination of both experimental and computational values. Both experimental [DOI:10.1021/ja048476w] and computational [DOI:10.1021/jp810475z] gas-phase acidities were taken from the literature. I selected the computed gas-phase acidities at the B3LYP/6-311G(d,p) level of theory as the corresponding computed gas-phase acidities for one of the experimentally determined values matched best between simulation and experiment. A linear regression was determined from three ligands with both known gas-phase acidities and Hammett substituent constants. The slope was −0.0110 ± 0.0009 and the intercept was 3.94 ± 0.35 with an R^2 value of 0.993. This model was used to predict the Hammett substituent constants of the two remaining alkoxide groups. These results are provided in Table S3G.

Table S3G: Data points used to estimate the Hammett substituent constants of two alkoxide ligands.

L – H	Gas-phase Acidity (D) $[\mathrm{kcal mol^{-1}}]$	Source	$\sigma\ (L)$	Source
CF_3CH_2OH	362.8	DOI:10.1021/jp810475z	-0.06	Predicted
CF_2HCH_2OH	370.5	DOI:10.1021/jp810475z	-0.15	Predicted
H_2O	390.3	DOI:10.1021/ja048476w	-0.37	DOI:10.1021/cr00002a004
CH_3OH	382.0	DOI:10.1021/ja048476w	-0.27	DOI:10.1021/cr00002a004
EtOH	378.3	DOI:10.1021/ja048476w	-0.24	DOI:10.1021/cr00002a004

1.2 *Influence of Proton Donor*

For modeling the influence of the proton donor, I only used data points for magnesium complexes that had the same ligands. I derived a linear model correlating the gas-phase acidity of the proton donors with the natural logarithm of the protolysis rate constant. The data points used to derive the linear model are provided in Table S4G. The linear correlation of the natural logarithm of the rate constant against tha gas-phase acidity of the proton donor yielded a slope of −0.016 ± 0.005 and an R^2 value of 0.91.

Table S4G: Data points used to model the proton donor influence.

M	L_1	L_2	L_3	D	A	k $[\mathrm{cm^3 s^{-1}}]$	Source	Gas-phase Acidity (D) $[\mathrm{kcal mol^{-1}}]$	Source
Mg	CH_3	CH_3COO	CH_3COO	H_2O	CH_3	1.20E-10	DOI:10.1021/ja048476w	390.3	DOI:10.1021/ja048476w
Mg	CH_3	CH_3COO	CH_3COO	CH_3OH	CH_3	1.31E-10	DOI:10.1021/ja048476w	382.0	DOI:10.1021/ja048476w
Mg	CH_3	CH_3COO	CH_3COO	EtOH	CH_3	1.47E-10	DOI:10.1021/ja048476w	378.3	DOI:10.1021/ja048476w

1.3 Influence of Acceptor

For modeling the influence of the acceptor, I only used data points for zinc complexes that had the same ligands and proton donors and only varied the acceptors. Importantly, as all the corresponding data points had two equivalent ligands of one type and one ligand of another type, there was an entropic advantage for phenyl to serve as the acceptor. This was corrected for by dividing the corresponding protolysis rate constants by 2 so that they are directly comparable to the protolysis rate constants where the one ligand of another type acts as acceptor. Additionally, there were only four pairs of data points where only the acceptor group was different. Hence, the change in the natural logarithm of the protolysis rate constant was correlated with the corresponding changes in the Hammett substituent constants. From the several pairs of data points several slope estimates were obtained which were subsequently averaged to obtain a final estimate. Notably, this resulted in relatively high uncertainty but the model was still decided to be used. An average slope for the change in the logarithm of the protolysis rate constant against the change in the Hammett substituent constant of -8.0 was obtained. Table S5G provides the data used to model the influence of the acceptor.

Table S5G: Data points used to model the influence of the acceptor.

M	L_1	L_2	L_3	D	A	k [cm^3s^{-1}]	Source	σ (A)	Source
Zn	Ph	Ph	4-OMe-Ph	CF_3CH_2OH	Ph	4.91E-12	Statistical Correction	-0.01	DOI:10.1021/cr00002a004
Zn	Ph	Ph	4-OMe-Ph	CF_3CH_2OH	4-OMe-Ph	9.16E-12	DOI:10.1021/acs.jpca.1c08964	-0.08	DOI:10.1021/cr00002a004
Zn	Ph	Ph	4-Me-Ph	CF_3CH_2OH	Ph	6.35E-12	Statistical Correction	-0.01	DOI:10.1021/cr00002a004
Zn	Ph	Ph	4-Me-Ph	CF_3CH_2OH	4-Me-Ph	8.86E-12	DOI:10.1021/acs.jpca.1c08964	-0.03	DOI:10.1021/cr00002a004
Zn	Ph	Ph	4-F-Ph	CF_3CH_2OH	Ph	3.21E-12	Statistical Correction	-0.01	DOI:10.1021/cr00002a004
Zn	Ph	Ph	4-F-Ph	CF_3CH_2OH	4-F-Ph	2.67E-12	DOI:10.1021/acs.jpca.1c08964	0.06	DOI:10.1021/cr00002a004
Zn	Ph	Ph	4-Cl-Ph	CF_3CH_2OH	Ph	2.31E-12	Statistical Correction	-0.01	DOI:10.1021/cr00002a004
Zn	Ph	Ph	4-Cl-Ph	CF_3CH_2OH	4-Cl-Ph	1.39E-12	DOI:10.1021/acs.jpca.1c08964	0.12	DOI:10.1021/cr00002a004

1.4 Test Set Predictions

To perform the test set predictions, the Hammett substituent for 2,4,6-trimethylphenyl was required. I obtained it from a recently developed web tool that is described in a preprint [DOI:10.26434/chemrxiv.14215964.v1]. The web tool is accessible at https://peter-ertl.com/molecular/substituents/sigmas.html. The value predicted for 2,4,6-trimethylphenyl was -0.04. All the models described in the previous sections were applied as additive correction to the reference data point for the organoferrate(II) anion provided. Additionally, when applicable, an entropic correction was applied to this reference data point to account for the change between the presence of three equivalent groups that could serve as acceptor and of only two equivalent ones. The parameters used for the predictions are provided in Tables S6G-8G and the corresponding prediction results in Table S9G.

Table S6G: Test set parameters of the ligands used for the predictions.

M	L_1	L_2	L_3	D	A	$\sigma_{mean}(L_i)$	Source
Fe	Ph	Ph	OCH_2CF_3	CF_3CH_2OH	Ph	-0.03	DOI:10.1021/cr00002a004
Fe	Ph	Ph	Ph	CF_2HCH_2OH	Ph	-0.01	DOI:10.1021/cr00002a004
Fe	Ph	Ph	OCH_2CF_2H	CF_2HCH_2OH	Ph	-0.06	DOI:10.1021/cr00002a004
Fe	$2,4,6-Me_3-Ph$	$2,4,6-Me_3-Ph$	$2,4,6-Me_3-Ph$	CF_3CH_2OH	$2,4,6-Me_3-Ph$	-0.04	DOI:10.26434/chemrxiv.14215964.v1
Fe	$2,4,6-Me_3-Ph$	$2,4,6-Me_3-Ph$	och2cf3	CF_3CH_2OH	$2,4,6-Me_3-Ph$	-0.05	DOI:10.26434/chemrxiv.14215964.v1

Table S7G: Test set parameters of the proton donors used for the predictions.

M	L_1	L_2	L_3	D	A	Gas-phase Acidity (D) [$kcal mol^{-1}$]	Source
Fe	Ph	Ph	OCH_2CF_3	CF_3CH_2OH	Ph	362.84	DOI:10.1021/jp810475z
Fe	Ph	Ph	Ph	CF_2HCH_2OH	Ph	370.46	DOI:10.1021/jp810475z
Fe	Ph	Ph	OCH_2CF_2H	CF_2HCH_2OH	Ph	370.46	DOI:10.1021/jp810475z
Fe	$2,4,6-Me_3-Ph$	$2,4,6-Me_3-Ph$	$2,4,6-Me_3-Ph$	CF_3CH_2OH	$2,4,6-Me_3-Ph$	362.84	DOI:10.1021/jp810475z
Fe	$2,4,6-Me_3-Ph$	$2,4,6-Me_3-Ph$	och2cf3	CF_3CH_2OH	$2,4,6-Me_3-Ph$	362.84	DOI:10.1021/jp810475z

Table S8G: Test set parameters of the acceptors used for the predictions.

M	L_1	L_2	L_3	D	A	σ (A)	Source
Fe	Ph	Ph	OCH_2CF_3	CF_3CH_2OH	Ph	-0.01	DOI:10.1021/cr00002a004
Fe	Ph	Ph	Ph	CF_2HCH_2OH	Ph	-0.01	DOI:10.1021/cr00002a004
Fe	Ph	Ph	OCH_2CF_2H	CF_2HCH_2OH	Ph	-0.01	DOI:10.1021/cr00002a004
Fe	$2,4,6-Me_3-Ph$	$2,4,6-Me_3-Ph$	$2,4,6-Me_3-Ph$	CF_3CH_2OH	$2,4,6-Me_3-Ph$	-0.04	DOI:10.26434/chemrxiv.14215964.v1
Fe	$2,4,6-Me_3-Ph$	$2,4,6-Me_3-Ph$	och2cf3	CF_3CH_2OH	$2,4,6-Me_3-Ph$	-0.04	DOI:10.26434/chemrxiv.14215964.v1

Table S9G: Final prediction results together with information about what models were used to correct the rate constant of the training data point.

M	L_1	L_2	L_3	D	A	k [$cm^3 s^{-1}$]	Source
Fe	Ph	Ph	OCH_2CF_3	CF_3CH_2OH	Ph	1.9E-11	Ligand Model
Fe	Ph	Ph	Ph	CF_2HCH_2OH	Ph	1.9E-11	Proton Donor Model
Fe	Ph	Ph	OCH_2CF_2H	CF_2HCH_2OH	Ph	2.5E-11	Ligand and Proton Donor Models
Fe	$2,4,6-Me_3-Ph$	$2,4,6-Me_3-Ph$	$2,4,6-Me_3-Ph$	CF_3CH_2OH	$2,4,6-Me_3-Ph$	4.3E-11	Ligand and Acceptor Models
Fe	$2,4,6-Me_3-Ph$	$2,4,6-Me_3-Ph$	och2cf3	CF_3CH_2OH	$2,4,6-Me_3-Ph$	3.2E-11	Ligand and Acceptor Models
